# An ethnobotanical survey of wild edible plants used by the Yi people of Liangshan Prefecture, Sichuan Province, China

**DOI:** 10.1186/s13002-019-0349-5

**Published:** 2020-02-26

**Authors:** Jing Wang, Barnabas C. Seyler, Tamara Ticktin, Yonggang Zeng, Kede Ayu

**Affiliations:** 1 School of Architecture and Civil Engineering, Chengdu University, Chengdu, 610106 China; 2 Department of Environment, Sichuan University, Chengdu, 610065 China; 3 Department of Botany, University of Hawai‘i at Mānoa, Honolulu, HI 96822 USA; 4 Leibo County Youth League Committee, Jincheng Town, 616550 Leibo County China

**Keywords:** Liangshan Yi Autonomous Prefecture, Yi people, Wild edible plants, Use values, Ethnobotany

## Abstract

**Background:**

Due to historical perceptions of Liangshan Yi Autonomous Prefecture (Sichuan Province, China) as being a violent place, and due to its rugged terrain, cultural differences, and relative inaccessibility, few researchers have conducted in-depth ethnobotanical investigations in Liangshan. But wild edible plants (WEPs) are widely consumed by the Yi people of Liangshan, and their associated ethnobotanical knowledge remains relatively unknown, especially outside of China. This study aimed to (1) investigate the WEPs used by the Liangshan Yi, (2) document the traditional knowledge held about these plants, (3) analyze their special preparation methods and consumption habits, and (4) identify species with important cultural significance to the Liangshan Yi.

**Methods:**

During 2016–2017, 396 Yi individuals were interviewed in 1 county-level city and 6 counties across Liangshan. Prior informed consent was obtained, and multiple ethnographic methods were utilized, including direct observation, semi-structured interviews, key informant interviews, informal discussions, and field visits. Market surveys were conducted in April, July, and August 2017 by interviewing 38 Yi merchants selling WEPs in 6 Liangshan traditional markets. We collected information about the parts consumed, preparation methods, consumption habits, growth pattern of species, collection months, market prices, and other uses of WEPs. Use values (UVs) were calculated to analyze the relative cultural importance of each WEP.

**Results:**

In total, 105 plant species belonging to 97 genera and 62 families were recorded. Rosaceae was the family with the largest number of species (14), and herbs (58 species) were the dominant growth form reported. Fruits (34 species), roots (21 species), and tender shoots (20 species) were the primary plant parts used for snacking and cooking. There were 6 main preparation and consumption methods of WEPs reported, ranging from primary food, famine food, snack, spice, culinary coagulant, and medicine, among a few other uses. The Liangshan Yi mainly collect WEPs from March to October, seldom collecting from November to February. There were 35 species of WEPs sold in the markets we visited in Liangshan. The price of medicinal plants was much higher than the price of food and fruits. In total, we documented 49 species of edible medicinal plants in Liangshan, accounting for 44.7% of all WEPs. They can be used for treating 27 medical conditions, including cough, diarrhea, injury, and headaches. The plants with the highest UVs were *Berberis jamesiana* (1.92), *Pyracantha fortuneana* (1.87), and *Artemisia capillaris* (1.44) indicating that these species are the most commonly used and important to the Liangshan Yi’s traditional life and culture.

**Conclusions:**

The traditional knowledge of WEPs from the accumulated experience of the Yi people’s long period residing in Liangshan reflects the cultural richness of the Yi and the plant diversity of the region. Future research on the nutrition, chemical composition, and bioactivity of the WEPs are needed. Some species with high medicinal value but with sharp wild population decline should be surveyed for resource assessment, conservation, and domestication potential.

## 摘要

背景:由于凉山彝族自治州(中国四川省)地形崎岖、交通不便, 文化制约、历史上民风彪悍, 很少有学者对凉山地区进行深入的民族植物学调查。凉山彝族采集食用多种野生食用植物(WEP_S_), 但其相关的民族植物学知识鲜为人知, 国外对此更是无从知晓。本研究的目的是:(1)对凉山彝族所食用的野生植物进行系统的研究; (2)记录这些植物的传统知识; (3)分析其特殊的制备方法和消费习惯; (4)识别对凉山彝族有重要文化意义的物种。

方法:在2016-2017年间, 对凉山州1个县级市和6个县的396名彝族人进行了访谈。采用了多种人类学方法, 包括直接观察、半结构式访谈、关键人物访谈、非正式讨论和田野调查。市场调查于2017年4月、7月和8月进行, 调查了凉山州6个传统集市, 并访问了38位彝族商贩。我们收集这些野生食用植物的利用部位、制备方法、利用方式, 生长模式、采集月份、市场价格和其他用途的相关信息。采用使用价值指数(UVs)来分析每个野生食用植物的的相对文化重要性。

结果:本研究共记录野生食用植物105种, 隶属于97属62科。其中蔷薇科植物种类最多(14种), 草本植物占优势(58种)。食用部位主要是果实(34种), 根(21种)和嫩枝(20种), 果实多作为零食食用, 根和嫩枝多需要进行烹饪后食用。凉山彝族制备和利用野生食用植物的方法可分为六类, 即, 日常饮食、饥荒食物、零食、调味品、食用凝固剂和药用植物。凉山彝族主要在3月至10月采集野生食用食物, 很少在11月至来年2月进行采集工作。调查发现有35种野生食用植物用于出售。药用植物的价格远高于食物和野果的价格。本研究共记录凉山地区食用药用植物49种, 占记录的野生食用植物总种数的44.67%, 它们可用于治疗27种疾病, 包括咳嗽、腹泻、受伤和头痛。使用价值(UVs)最高的植物是川滇小檗(1.92)、火棘(1.87)和茵陈蒿(1.44), 表明这些植物是凉山彝族传统生活和文化中最常用和最重要的种类。

结论:凉山彝族利用野生食用植物的传统知识源于在当地长期居住积累的生活经验, 反映了凉山彝族文化的丰富性和该地区植物的多样性。未来可对这些野生食用植物的的营养成分、化学成分和生物活性进行进一步的研究; 对一些具有较高药用价值但野生种群数量急剧下降的物种应进行资源评估、保护和驯化潜力调查研究。

## Introduction

Adapting to continued human population growth and global climate change requires a diversity of food plants to ensure a safe and resilient food supply [1–7]. In particular, wild edible plants (WEPs) are of great significance in maintaining the productivity and stability of traditional agro-ecosystems [5, 8]. In times of famine and scarcity, these sources of nutrients and health-promoting compounds have received heightened attention in rural and suburban areas [9, 10]. WEPs remain essential components of the diets for many people in developing countries, especially in periods of seasonal food shortage [11]. Consequently, conserving WEPs is necessary to ensure the ongoing supply of diverse genetic resources that are critical to global food security [2, 4, 5, 12].

As living standards rise, there is also an increasing global demand for healthier and safer food [13]. Compared with cultivated vegetables, WEPs require less maintenance, are not dependent on chemical fertilizers or pesticides, and are richer sources of micronutrients [5]. Some WEPs have also been described as “functional foods” because they contain physiologically active ingredients capable of providing health benefits beyond basic nutrition [10]. Multiple authors have noted a continuum in many cultures between “food” and “medicine” plants, with species initially selected as medicine later used primarily for food (or vice versa), and the concepts of *food* and *medicine* themselves are infrequently differentiated, instead taken together as synonymous with “healthy eating” [9, 14–17]. Furthermore, whether a particular plant is perceived as a food, medicine, or even poison often depends on the part used or quantity ingested, as well as how and when it is collected/prepared [9, 16].

Since local cultures select edible species over time based on many years of experience, traditional ethnobotanical knowledge and associated practices about WEPs are highly dependent on the local context [10]. It is, therefore, increasingly important to carry out systematic ethnobotanical investigations to document WEPs utilized in rural communities and by the socio-cultural groups that are dependent upon them [18]. Fortunately, in recent decades, focused studies of WEPs have proliferated worldwide, including in Africa [6, 19, 20], South Asia [4, 21], East Asia [5, 22, 23], Europe [10, 24], North America [17], South America [7, 11], and Oceania [25]. Nevertheless, the WEPs utilized by many unique socio-cultural groups in each of these diverse geographic regions remain understudied. Yet, in addition to documenting WEPs around the world, studies should also strive for theoretical rigor by testing hypotheses associated with the use, selection, and perception of WEPs in the local communities [26].

Liangshan Yi Autonomous Prefecture (Liangshan) is a mountainous rural jurisdiction in southwest Sichuan Province, China. It is the single largest settlement area for the Yi minority people in China, being the primary home of the most populous and geographically most widely distributed branch of Yi [27, 28]. For many centuries, Liangshan was considered by outsiders to be an especially dangerous place, with violent clashes and open warfare between the Yi and Han Chinese populations [29]. Liangshan was the last significant region of China to resist Communist control, so the people living there largely governed their own affairs until the Communist penetration of the area began in the 1950s. Before the 1956 reforms, the Yi people of Liangshan maintained a rigid slave-based feudal society, raiding neighboring Han communities and subjugating travelers as slaves. Despite the formal abolition of the system in the 1950s, the hierarchical classes, associated values systems, and other unique cultural characteristics have remained, largely intact, to this day [30]. Consequently, due to external perceptions about its remoteness, rugged terrain, cultural considerations, and relative inaccessibility, few ethnobotanists have conducted in-depth investigations in Liangshan.

Of note, few studies have specifically analyzed the WEPs harvested by the Liangshan Yi, and the only in-depth studies of this kind (all published in Chinese) have documented plants used for food by the Yi in a single county of Liangshan [31], as well as plants used for dye [32] and folk customs [33]. The richness of Liangshan’s traditional culture and biodiversity warrants deeper investigations. Therefore, this study sought to identify the WEPs used by Liangshan’s Yi people, document their uses, the plant parts used, and the traditional knowledge held about these plants. We also sought to assign plant use value scores to the WEPs utilized by the Liangshan Yi to identify the culturally most important taxa, which will help prioritize plants for conservation purposes. Based on the composition of the local flora, and because family tends to be a strong predictor of plant use value [26, 34], we hypothesized that (1) certain families (Compositae, Lamiaceae, Rosaceae) would have more species with higher use values as a result of their (a) greater abundance in Liangshan’s native flora and (b) global importance for food crop species. Although other globally important food-crop families (e.g., Solanaceae) are now commonly cultivated by the Liangshan Yi, they are relatively less common among the local flora. We also hypothesized that (2) fruit would be the plant part most frequently used, due to the local abundance of edible fruiting trees in the local flora; (3) most plants would be harvested during summer and fall, due to the seasonality of fruit maturation; and (4) given that there is no clear distinction in many cultures between the concepts of *food* and *medicine*, some WEPS would also have medicinal value.

## Materials and methods

### Liangshan’s ecology and climate

Liangshan Prefecture encompasses 60,423 km^2^ and, located in the southwest of Sichuan Province, lies between 26° 03′ to 29° 18′ N latitude and 100° 03′ to 103° 52′2E longitude. The region has a subtropical monsoon climate, with warm winters, dry springs, and ample sunshine year-round. The rainy monsoon conditions with passing clouds moderate the summer temperatures as well, with an average annual temperature of 16–17 °C. To the east, west, and south, Liangshan is surrounded on three sides by the Jinsha River, and, to the north, it is bounded by the Dadu River, thus forming a relatively closed geographical unit. With the Greater and Lesser Xiangling Mountains as the boundary, there is a distinct contrast of climates in Liangshan with dryer conditions to the south and west and moister conditions to the north and east.

Liangshan also constitutes the northern section of the Hengduan Mountain Range, which serves as a topographic bridge between the Sichuan Basin and the Yunnan-Guizhou Plateau [35], forming the core area of the Eastern Himalayan Biodiversity Hot Spot [36]. Consequently, Liangshan is dominated by mountains and plateaus, accounting for more than 90% of the entire area, with the remaining 10% being hills, basins, and plains. The altitude ranges from 325 m below sea level up to 5958 m above sea level. These elevational extremes, with warmer temperatures prevailing at lower altitudes and cooler conditions at higher altitudes, also create a wide diversity of microclimates in close proximity, with a great abundance of plant species and high endemism. The vegetation cover of Liangshan includes more than 2 million hectares of woodlands and grasslands with more than 4000 plant species [37]. Due to its rich plant resources, the people living in the region for generations, particularly the Yi, have accumulated a wealth of ethnobotanical knowledge [33, 38].

### Liangshan demographics, language, and study site locations

The Yi comprise the seventh largest ethnic group in China, with a total population of about 9 million people primarily spread across the southwest Chinese provinces of Yunnan, Sichuan, and Guizhou. The Yi account for the majority of Liangshan’s total population, with the 2016 census indicating that of Liangshan’s 5,117,825 people, 2,647,791 were Yi (51.7%), followed by the Han (44.8%), Tibetan (1.41%), Mongolian (0.64%), and Hui (0.42%), among a few others [39]. In total, Liangshan’s population consists of 14 ethnocultural groups with an estimated density of 0.85 people per square kilometer [40].

**Fig. 1:**
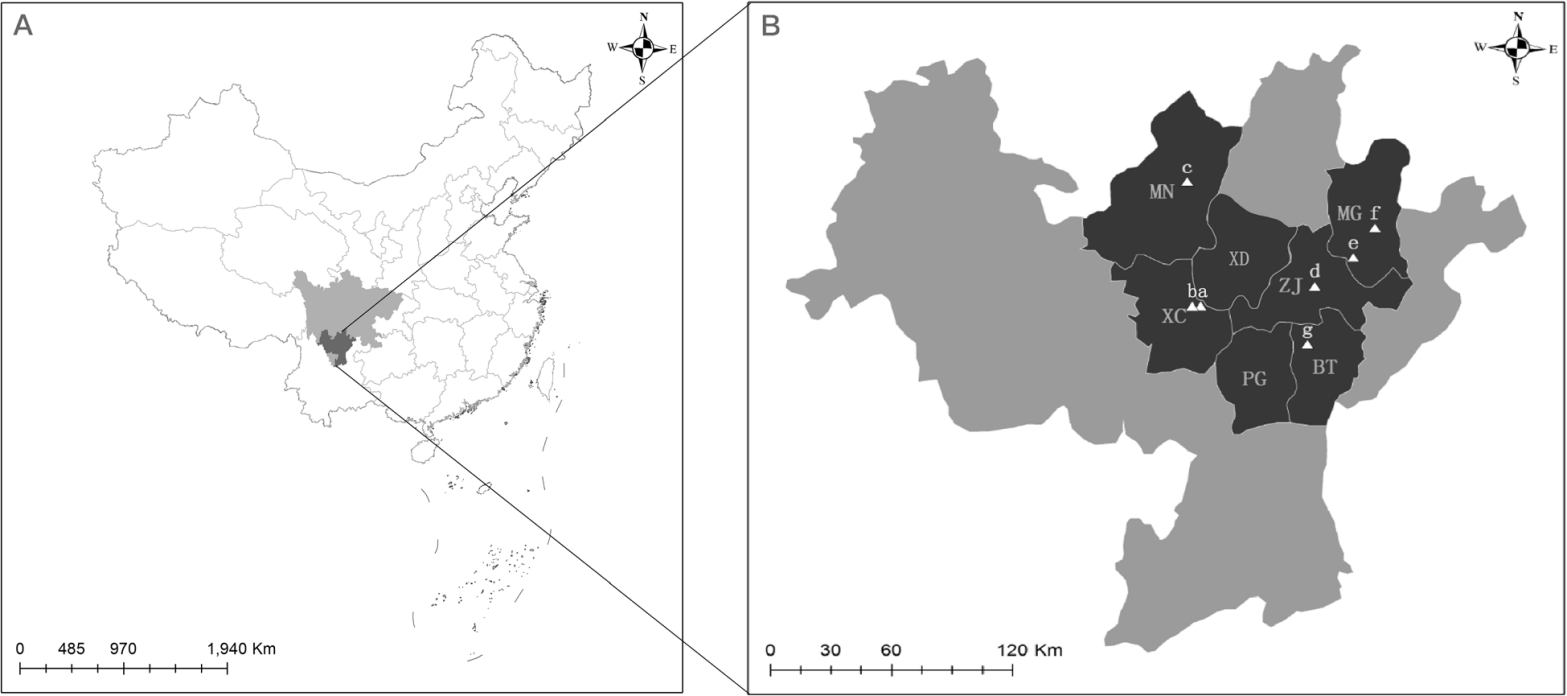
Location of the study areas. **a** Location of Liangshan Prefecture (dark gray) within Sichuan Province (light gray) and China (white). **b** Location of the field survey jurisdictions (dark gray) within Liangshan (light gray), with market locations indicated by triangles. BT, Butuo County; MG, Meiggu County; MN, Mianning County; PG, Puge County; XC, Xichang City; XD, Xide County; ZJ, Zhaojue County. (a) Xichang Binhe Market; (b) Chang’an Village Farmers Market, Xichang City; (c) Mianning County Farmers Market; (d) Zhaojue Comprehensive Farmers Market; (e) Jiukou Township Market, Meigu County; (f) Meigu County Farmers Market; (g) Butuo County Farmers Market

Administratively, Liangshan is comprised of 16 counties and one county-level city (Xichang City), which serves as the prefectural capital. Prior to 1978, Zhaojue County served as Liangshan’s capital. The Liangshan Yi belong to the northern dialect branch of the Yi (*Nuosu*) language, which is further subdivided into 4 dialects; these are often referred in published literature in Chinese as *Sheng-zha* (圣乍), *Yi-nuo* (义诺), *Suo-di* (所地), and *A-du* (阿都) [28, 41, 42]. We chose study sites in six counties and Xichang City, representative of the geographic and cultural diversity of the Liangshan Yi (Fig. 1, Table 1). The *Sheng-zha* dialect (*Shyp nra hxop*: ), based in Xide County and also widely spoken in Mianning County, Xichang City, and Zhaojue County, is considered the prestige or standard pronunciation for the Yi Language [41–43]. Butuo County represents the *A-du* dialect (*A dur hxop*: ), Meigu County represents the *Yi-nuo* dialect (*Yyp nuo hxop*: ), and Puge County represents the *Suo-di* dialect (*Suo ndip hxop*: ). All *Nuosu* words in this paper and supporting materials follow the official Yi language phonetic alphabet, affixing a consonant symbol at the end of certain syllables to represent tones: (1) “t” for a high, flat register, (2) “x” for a mid to high register (mid-rising tone), and (3) “p” for a low-falling register. There is no mark for the mid-register tone [28, 44].

**Table 1:** Study site locations

County/city name	Fieldwork months	Area (ha)	Total population	Yi population	Percentage (Yi/total)	Annual per capita income (RMB)	Participants (F/M)	Market survey location (no. of participants)	Latitude and longitude (market)
Butuo	Apr., Jul., Aug., Oct. 2017	1685	191,213	184,128	96	¥6386	74 (33/41)	Butuo County Farmers Market (6)	102.8109, 27.7078
Meigu	Apr., Jul., Aug. 2017	2515	268,739	265,689	99	¥6246	63 (32/31)	Jiukou Township Market, Meigu County (3); Meigu County Farmers Market (5)	103.0252, 28.1722; 103.1307, 28.3311
Mianning	Jul., Aug. 2016; Apr., Jul., Aug. 2017	4422	398,071	160,098	40	¥11,156	73 (38/35)	Mianning County Farmers Market (8)	102.1761, 28.5518
Puge	Oct., Nov., Dec. 2016	1905	198,982	165,786	83	¥7454	18 (7/11)		
Xichang	Apr. 2017	2657	652,947	126,493	19	¥13,620	19 (5/14)	Xichang Binhe Market (8); Chang’an Village Farmers Market, Xichang City (2)	102.2697, 27.8948; 102.2284, 27.8917
Xide	Oct., Nov., Dec., 2016; Jul., Aug. 2017	2202	229,690	208,604	91	¥6347	57 (26/31)		
Zhaojue	Jul., Aug. 2017	2702	314,461	308,555	98	¥6675	92 (45/47)	Zhaojue Comprehensive Farmers Market (6)	102.8374, 28.0157
						Total	396 (186/210)		

### Yi traditional culture

The ancestors of the Liangshan Yi are believed to be the two ancient tribes of Guhou and Qunei, who once lived in Zizipuwu (Zhaotong area) of what is now Yunnan Province. They moved into Liangshan more than 2000 years ago, and their descendants gradually differentiated into the several tribes that are now spread throughout the Liangshan region [45]. Due to the historic physical and social isolation of the area, the Yi culture of Liangshan is quite different from the Yi living outside of Sichuan (other than a few border areas, including Ninglang County and Zhaotong in northern Yunnan), which have been much more deeply influenced by Han culture and religion [27, 28, 33, 46, 47].

The Liangshan Yi primarily subscribe to polytheistic animism, believing that their ancestors, spirits, and ghosts are able to influence the health of people, the success of their clan, the bounty of the harvest, the fertility of cattle, and the harmony of the community. Rituals serve as the main vehicle for the expression of their beliefs and traditional sentiments, being the primary means for balancing and adjusting the relationships between humans and supernatural beings. The Yi ritual specialists and traditional practitioners are called *bimox* () and *sunyit* (), respectively [48]. Throughout these ceremonies, some WEPs are used for both the ritual concoctions themselves as well as for offerings.

### Field survey and data collection

Between July 2016 and September 2017, we conducted field ethnobotanical surveys in the 7 jurisdictions (6 counties and 1 city) across Liangshan (Table 1). Following snowball sampling methods [49], we interviewed a total of 396 local Yi, of which 195 were female and 201 were male. Participants were between the ages of 12 and 84. The purpose of the study was briefly explained to each, and informed consent was obtained orally. Ethnobotanical data were collected using different ethnographic methods (direct observation, semi-structured interviews, key informant interviews, informal discussions, and field visits) with the assistance of native Yi language translators. Interviews took place in a location of each participant’s choice, often being their homes but sometimes in the field. Following established interview protocols [20, 49–51], participants were first asked to name the WEPs that they gather, then asked follow-up questions about the parts consumed, preparation methods, consumption habits, growth pattern of the species, collection months, and other uses of the WEPs. Demographic variables of each participant were also collected at the end of each interview, including their name, age, sex, level of education, and occupation.

During the investigation, we also conducted market surveys in April, July, and August 2017, interviewing 38 Yi merchants who sold WEPs in 7 traditional markets within 5 of the jurisdictions (Table 1, Fig. 1). We asked the same questions as those in the field interviews, including about WEPs that they regularly collect/sell even if they were not available at the time of the interview, and we also recorded their demographic variables. For the WEPs that were currently being sold at each stall, we recorded the prices of each.

We collected herbarium voucher specimens during field walks with participants, with initial identification being conducted on-site. Voucher specimens were later identified by J.W. and deposited in the Environmental Laboratory of Chengdu University (Table 2). Identification was carried out using keys, online plant databases, pictorial floras, plant dictionaries, and other taxonomic references, with accepted Latin names verified using The Plant List (www.theplantlist.org).

**Table 2:** The WEPs traditionally used by the Liangshan Yi

Scientific name	Family name	Growth form	Part used (consumption pattern)	Medicinal uses(s)	Collection months	Sold as/price (¥/kg)	ΣUs	UVs	UV rank	Voucher no.
*Actinidia kolomikta* (Rupr. & Maxim.) Maxim.	Actinidiaceae	Climber	Fruit (snack)		9–10	N	244	0.62	51	LS0081
*Sambucus adnata* Wall. ex DC.	Adoxaceae	Herb	Fruit (snack), aboveground part (medicine, other use)	Bone fracture, rheumatism	1–12	N	422	1.07	10	LS0152
*Sambucus williamsii* Hance	Adoxaceae	Shrub	Leaf, bark (medicine)	Bone fracture	1–12	N	207	0.52	62	LS0181
*Viburnum betulifolium* Batalin	Adoxaceae	Shrub	Fruit (snack, spice)		9–10	N	249	0.63	49	LS0125
*Amaranthus blitum* L.	Amaranthaceae	Herb	Tender shoot (primary food)		3–6	N	233	0.59	54	LS0166
*Celosia argentea* L.	Amaranthaceae	Herb	Seed (famine food)		7–10	N	99	0.25	99	LS0093
*Chenopodium hybridum* L.	Amaranthaceae	Herb	Tender shoot (primary food)		4–6	N	287	0.72	38	LS0016
*Allium macrostemon* Bunge	Amaryllidaceae	Herb	Whole plant (primary food, medicine, spice)	Gastropathy	3–11	N	228	0.58	55	LS0147
*Allium ovalifolium* Hand.-Mazz.	Amaryllidaceae	Herb	Leaf (primary food)		3–10	N	131	0.33	90	LS0177
*Toxicodendron vernicifluum* (Stokes) F.A. Barkley	Anacardiaceae	Tree	Tender shoot (primary food), branch (other use)		3–5	N	213	0.54	61	LS0140
*Angelica sinensis* (Oliv.) Diels	Apiaceae	Herb	Root (primary food, medicine)	Tonify	6–9	Medicine/40 (wet)	371	0.94	15	LS0043
*Oenanthe javanica* (Blume) DC.	Apiaceae	Herb	Aboveground part (primary food)		3–6	N	258	0.65	45	LS0038
*Metaplexis japonica* (Thunb.) Makino	Apocynaceae	Climber	Fruit (snack)		8–10	N	130	0.33	91	LS0032
*Aralia chinensis* L.	Araliaceae	Tree	Tender shoot (primary food), branch (other use)		3–5	Food/10	269	0.68	41	LS0375
*Aristolochia versicolor* S.M.Hwang	Aristolochiaceae	Climber	Root (medicine)	Headache, injury, gastroenteritis	4–10	Medicine/200	52	0.13	105	LS0193
*Polygonatum cyrtonema* Hua	Asparagaceae	Herb	Root (primary food, medicine)	Tonify	5–8	Medicine/40	334	0.84	29	LS0536
*Begonia grandis* subsp. *sinensis* (A.DC.) Irmsch.	Begoniaceae	Herb	Stem (primary food)		4–10	N	140	0.35	85	LS0151
*Berberis jamesiana* Forrest & W.W.Sm.	Berberidaceae	Shrub	Fruit (culinary coagulant, snack, spice), root (medicine)	Diarrhea	9–12	N	761	1.92	1	LS0248
*Mahonia bealei* (Fortune) Pynaert	Berberidaceae	Shrub	Fruit (snack), bark and root (medicine)	Diarrhea	1–12	Medicine/50	204	0.52	65	LS0062
*Incarvillea diffusa* Royle	Bignoniaceae	Herb	Aboveground part (medicine)	Hepatitis	1–12	N	214	0.54	60	LS0558
*Cynoglossum amabile* Stapf & J.R.Drumm.	Boraginaceae	Herb	Root (medicine)	Hemorrhoid, enteritis	3–10	N	198	0.50	68	LS0013
*Capsella bursa-pastoris* (L.) Medik.	Brassicaceae	Herb	Tender shoot (primary food)		3–5	N	155	0.39	84	LS0423
*Cardamine tangutorum* O.E.Schulz	Brassicaceae	Herb	Tender shoot (primary food, medicine)	Hypertension	3–5	Food/10	273	0.69	39	LS0279
*Nasturtium officinale* R.Br.	Brassicaceae	Herb	Aboveground part (primary food)		4–10	Food/5	139	0.35	87	LS0063
*Rorippa dubia* (Pers.) H.Hara	Brassicaceae	Herb	Tender shoot (primary food)		3–6	N	107	0.27	95	LS0121
*Hylocereus undatus* (Haw.) Britton & Rose	Cactaceae	Shrub	Flower (primary food)		7–11	N	98	0.25	100	LS0162
*Opuntia ficus-indica* (L.) Mill.	Cactaceae	Shrub	Stem (primary food, medicine), fruit (snack)	Tonsillitis	1–12	N	294	0.74	37	LS0346
*Codonopsis pilosula* subsp. *tangshen* (Oliv.) D.Y.Hong	Campanulaceae	Herb	Root (primary food, medicine)	Tonify, gallstone	4–8	Medicine/50	342	0.86	27	LS0223
*Leycesteria formosa* Wall.	Caprifoliaceae	Shrub	Tender shoot (medicine)	Measles	3–10	N	132	0.33	89	LS0509
*Arctium lappa* L.	Compositae	Herb	Root (primary food, medicine)	Tonify, detoxify	3–5	Medicine/20	382	0.96	14	LS0315
*Artemisia capillaris* Thunb.	Compositae	Herb	Tender shoot (famine food), aboveground part (medicine, other use)	Injury	3–10	N	569	1.46	3	LS0256
*Cirsium shansiense* Petr.	Compositae	Herb	Root (primary food, medicine)	Tonify, nephrosis	3–11	Medicine/12	452	1.14	7	LS0362
*Eclipta prostrata* (L.) L.	Compositae	Herb	Whole plant (medicine)	Diarrhea, cough, pneumonia	3–10	N	168	0.42	79	LS0033
*Kalimeris indica* (L.) Sch.Bip.	Compositae	Herb	Tender shoot (primary food), root (medicine)	Diarrhea	3–10	N	140	0.35	86	LS0255
*Pseudognaphalium affine* (D.Don) Anderb.	Compositae	Herb	Flower (famine food), whole plant (other use)		2–5	N	190	0.48	71	LS0014
*Sonchus oleraceus* (L.) L.	Compositae	Herb	Tender shoot (primary food)		3–6	N	201	0.51	67	LS0463
*Taraxacum mongolicum* Hand.-Mazz.	Compositae	Herb	Leaf (primary food), whole plant (medicine)	Cough	3–10	Medicine/5	334	0.84	30	LS0410
*Cornus kousa* subsp. *chinensis* (Osborn) Q.Y.Xiang	Cornaceae	Tree	Fruit (snack)		9–10	Snack/10	196	0.49	70	LS0407
*Trichosanthes kirilowii* Maxim.	Cucurbitaceae	Climber	Flower (medicine)	Cough	5–8	N	114	0.29	94	LS0058
*Araiostegia divaricata* var. *formosana* (Hayata) M. Kato	Davalliaceae	Herb	Root (medicine)	Hypertension	3–10	N	67	0.17	104	LS0579
*Pteridium aquilinum* (L.) Kuhn	Dennstaedtiaceae	Herb	Tender shoot (primary food), root (famine food)		3–6	Food/10	536	1.35	4	LS0004
*Dioscorea polystachya* Turcz.	Dioscoreaceae	Climber	Root (primary food), bulbil (snack)		3–10	N	329	0.83	33	LS0376
*Diospyros lotus* L.	Ebenaceae	Tree	Fruit (snack)		10–11	N	170	0.43	78	LS0467
*Elaeagnus pungens* Thunb.	Elaeagnaceae	Shrub	Fruit (snack)		8–9	N	346	0.87	23	LS0221
*Equisetum giganteum* L.	Equisetaceae	Herb	Whole plant (medicine)	Cold, headache, stomachache	3–10	N	102	0.26	97	LS0303
*Vaccinium fragile* Franch.	Ericaceae	Shrub	Fruit (snack)		7–10	N	224	0.57	57	LS0452
*Eucommia ulmoides* Oliv.	Eucommiaceae	Tree	Bark (primary food, medicine)	Nephropathy	1–12	N	178	0.45	76	LS0199
*Quercus schottkyana* Rehder & E.H.Wilson	Fagaceae	Tree	Seed (snack)		10–11	N	156	0.39	83	LS0366
*Helwingia japonica* (Thunb.) F.Dietr.	Helwingiaceae	Shrub	Leaf (primary food)		3–6	N	105	0.27	96	LS0319
*Iris forrestii* Dykes	Iridaceae	Herb	Root (medicine)	Cough	3–10	N	178	0.45	77	LS0417
*Mentha canadensis* L.	Lamiaceae	Herb	Tender shoot (primary food, medicine, spice)	Hyperthermia	3–10	Spice/10	393	0.99	12	LS0403
*Perilla frutescens* (L.) Britton	Lamiaceae	Herb	Tender shoot (primary food), seed (Spice)		8–10	N	438	1.11	8	LS0292
*Akebia trifoliata* (Thunb.) Koidz.	Lardizabalaceae	Climber	Fruit (snack)		7–8	N	236	0.60	52	LS0481
*Decaisnea insignis* (Griff.) Hook.f. & Thomson	Lardizabalaceae	Shrub	Fruit (snack)		10–11	N	167	0.42	80	LS0200
*Litsea cubeba* (Lour.) Pers.	Lauraceae	Tree	Fruit and root (spice)		7–10	Spice/40	205	0.52	64	LS0561
*Litsea pungens* Hemsl.	Lauraceae	Tree	Fruit and root (spice)		7–10	Spice/40	343	0.87	25	LS0535
*Pueraria montana* var. *lobata* (Willd.) Sanjappa & Pradeep	Leguminosae	Climber	Root (snack)		7–10	Snack/20	183	0.46	74	LS0448
*Spatholobus suberectus* Dunn	Leguminosae	Climber	Stem (medicine)	Heart disease	3–10	Medicine/40	89	0.22	102	LS0457
*Vicia sativa* L.	Leguminosae	Herb	Tender shoot (primary food)		1–12	N	189	0.48	72	LS0252
*Fritillaria cirrhosa* D.Don	Liliaceae	Herb	Bulb (medicine)	Cough, injury	7–8	Medicine/ 2000(dry)	261	0.66	44	LS0471
*Huperzia squarrosa* (G. Forst.) Trevis.	Lycopodiaceae	Herb	Whole plant (medicine)	Rheumatism, gastropathy	3–10	Medicine/50	78	0.20	103	LS0532
*Lycopodium japonicum* Thunb.	Lycopodiaceae	Herb	Spore powder (medicine)	Rheumatism	3–10	Medicine/200	95	0.24	101	LS0441
*Malva verticillata* L.	Malvaceae	Herb	Whole plant (medicine)	Delivery	1–12	N	270	0.68	40	LS0022
*Paris polyphylla* Sm.	Melanthiaceae	Herb	Root (medicine)	Muscle pain, injury	3–10	Medicine/600	254	0.64	46	LS0424
*Toona sinensis* (Juss.) M.Roem.	Meliaceae	Tree	Tender shoot (primary food, medicine)	Diarrhea	3–5	N	354	0.89	21	LS0369
*Ficus pumila* L.	Moraceae	Climber	Fruit (snack)		6–8	N	115	0.29	93	LS0161
*Ficus tikoua* Bureau	Moraceae	Shrub	Fruit (snack)		7–8	N	295	0.74	36	LS0148
*Morus australis* Poir.	Moraceae	Tree	Fruit (snack)		4–5	N	253	0.64	47	LS0389
*Musa basjoo* Siebold & Zucc. ex Iinuma	Musaceae	Tree	Flower (medicine), fruit(snack)	Heart disease	1–12	N	207	0.52	63	LS0098
*Myrica nana* A. Chev.	Myricaceae	Shrub	Fruit (snack)		6–8	Snack/10	198	0.50	69	LS0225
*Matteuccia struthiopteris* (L.) Tod.	Onocleaceae	Herb	Tender shoot (primary food)		3–5	Food/10	345	0.87	24	LS0272
*Ophioglossum vulgatum* L.	Ophioglossaceae	Herb	Whole plant (primary food, medicine)	Tonify	3–10	Medicine/200	321	0.81	34	LS0446
*Bulbophyllum odoratissimum* (Sm.) Lindl. ex Wall.	Orchidaceae	Herb	Whole plant (medicine)	Cough	7–10	Medicine/40	100	0.25	98	LS0569
*Gastrodia elata*	Orchidaceae	Herb	Rhizome (primary food, medicine)	Headache	3–6	Medicine/100 (wet), 500 (dry)	430	1.09	9	LS0142
*Osmunda japonica* Thunb.	Osmundaceae	Herb	Tender shoot (primary food)		3–5	N	332	0.84	32	LS0367
*Oxalis corniculata* L.	Oxalidaceae	Herb	Aboveground part (snack, spice, other use)		1–12	N	461	1.16	6	LS0343
*Plantago major* L.	Plantaginaceae	Herb	Aboveground part (primary food, medicine)	Diarrhea, cough	3–10	Medicine/5	416	1.05	11	LS0007
*Fargesia spathacea* Franch.	Poaceae	Shrub	Tender shoot (primary food)		3–5	Food/14	357	0.90	17	LS0087
*Imperata cylindrica* (L.) Raeusch.	Poaceae	Herb	Root (primary food, medicine)	Nosebleed, cough	3–10	N	295	0.74	35	LS0259
*Reynoutria multiflora* (Thunb.) Moldenke	Polygonaceae	Herb	Root (medicine), leaf (culinary coagulant)	Headache	1–12	Medicine/40	222	0.56	58	LS0549
*Lemmaphyllum carnosum* (J. Sm. ex Hook.) C. Presl	Polypodiaceae	Herb	Aboveground part (medicine)	Cough, injury	3–10	N	163	0.41	81	LS0349
*Pyrrosia lingua* (Thunb.) Farw.	Polypodiaceae	Herb	Whole plant (medicine)	Gallstone	3–10	N	138	0.35	88	LS0141
*Lysimachia congestiflora* Hemsl.	Primulaceae	Herb	Whole plant (medicine)	Gallstone	3–10	N	220	0.56	59	LS0172
*Anemone vitifolia* Buch.-Ham. ex DC.	Ranunculaceae	Herb	Fruit (famine food)		9–12	N	263	0.66	43	LS0328
*Hovenia dulcis* Thunb.	Rhamnaceae	Tree	Infructescence shaft (snack, medicine)	Tonify	8–10	Snack/14	204	0.52	66	LS0300
*Agrimonia pilosa* Ledeb.	Rosaceae	Herb	Aboveground part (medicine)	Diarrhea	1–12	N	179	0.45	75	LS0224
*Crataegus scabrifolia* (Franch.) Rehder	Rosaceae	Tree	Fruit (snack, medicine)	Cough	8–10	N	354	0.89	20	LS0179
*Duchesnea indica* (Jacks.) Focke	Rosaceae	Herb	Fruit (snack)		6–10	N	363	0.92	16	LS0427
*Fragaria nilgerrensis* Schltdl. ex J.Gay	Rosaceae	Herb	Fruit (snack)		5–9	N	356	0.90	18	LS0124
*Potentilla discolor* Bunge	Rosaceae	Herb	Whole plant (medicine)	Diarrhea, gastropathy	3–10	N	245	0.62	50	LS0385
*Prunus trichostoma* Koehne	Rosaceae	Tree	Fruit (snack)		7–10	N	225	0.57	56	LS0180
*Pyracantha fortuneana* (Maxim.) H.L.Li	Rosaceae	Shrub	Fruit (famine food, snack, other use)		1–12	N	741	1.87	2	LS0404
*Pyrus pashia* Buch.-Ham. ex D.Don	Rosaceae	Tree	Fruit (snack)		9–10	N	340	0.86	28	LS0483
*Rosa omeiensis* Rolfe	Rosaceae	Herb	Fruit (snack)		5–8	Snack/10	385	0.97	13	LS0337
*Rosa roxburghii* Tratt.	Rosaceae	Shrub	Fruit (snack)		8–10	Snack/10	236	0.60	53	LS0021
*Rubus ellipticus* var. *obcordatus* (Franch.) Focke	Rosaceae	Shrub	Fruit (snack)		4–5	N	333	0.84	31	LS0055
*Rubus inopertus* (Focke) Focke	Rosaceae	Shrub	Fruit (snack)		7–8	N	343	0.87	26	LS0023
*Rubus mesogaeus* Focke	Rosaceae	Shrub	Fruit (snack)		7–8	N	348	0.88	22	LS0566
*Rubus wallichianus* Wight & Arn.	Rosaceae	Shrub	Fruit (snack)		5–6	N	356	0.90	19	LS0035
*Houttuynia cordata* Thunb.	Saururaceae	Herb	Whole plant (primary food, medicine), root (spice)	Dyspepsia	3–10	Food/6	500	1.26	5	LS0056
*Schisandra rubriflora* Rehder & E.H.Wilson	Schisandraceae	Climber	Fruit (snack, medicine)	Tonify	9–12	N	264	0.67	42	LS0123
*Smilax stans* Maxim.	Smilacaceae	Shrub	Tender hoot (primary food)		4–8	Food/16	162	0.41	82	LS0127
*Physalis alkekengi* L.	Solanaceae	Herb	Fruit (snack)		6–10	N	128	0.32	92	LS0130
*Vitis heyneana* Roem. & Schult*.*	Vitaceae	Climber	Fruit (snack)		6–10	N	187	0.47	73	LS0017
*Hemerocallis citrina* Baroni	Xanthorrhoeaceae	Herb	Flower (primary food)		5–8	N	251	0.63	48	LS0500

### Data analysis

We grouped all WEPs into the following seven (non-exclusive) categories based on consumption pattern: primary food, famine food, snack, spice, culinary coagulant, medicine, and other uses. All data on participant demographics and the WEPs they identified were entered into a Microsoft Excel spreadsheet and organized for statistical analysis. Following the methods of Regassa et al. [20], we calculated the descriptive statistics on the number and percentage of species, genera, and families of WEPs, as well as their growth forms and the parts consumed. We calculated the use values for each species of WEP. The formula we used was adapted from Phillips and Gentry [52] by first considering a single participant interview [11]:

$$ {\mathrm{U}\mathrm{V}}_s=\varSigma {\mathrm{U}}_s/n $$

Where UV_*s*_ refers to the use value of a particular species “s,” *n* is the total number of respondents in the sample *(n* = 396), and *U*_*s*_ refers to the number of citations of use mentioned by each participant for a particular species “s.” The use values for each species were compiled into a table for interpretation [11].

## Results

### Taxonomic diversity of WEPs

The interview participants reported 105 WEPs from 97 genera and 62 families (Table 2). The families with the largest representation were Rosaceae (14 species), followed by Compositae (8 species), Brassicaceae (4 species), and Adoxaceae, Amaranthaceae, Leguminosae, and Moraceae (3 species each). Amaryllidaceae, Apiaceae, Berberidaceae, Cactaceae, Lamiaceae, Lardizabalaceae, Lauraceae, Lycopodiaceae, Orchidaceae, Poaceae, and Polypodiaceae each had 2 species. The remaining 44 families were represented by a single species each. The majority of WEPs were herbs (58 species), followed by shrubs (21 species), trees (15 species), and climbing plants (11 species).

Due to the dialectal diversity in Liangshan and across our site locations, while documenting the WEPs utilized by the Liangshan Yi, we also documented multiple local names for certain species (Table 3).

**Table 3:** Comparison of names used for WEPs across Liangshan. Alternative spellings for plant names from the same dialect are given in parentheses

Scientific name	*Nuosu* (Yi language) names: dialects and representative counties								Chinese name	
	*Sheng-zha* dialect (Xide County)	Characters	*A-du* dialect (Butuo County)	Characters	*Suo-di* dialect (Puge County)	Characters	*Yi-nuo* dialect (Meigu County)	Characters	Romanized pinyin	Characters
*Actinidia kolomikta* (Rupr. & Maxim.) Maxim.	ce le mop ce ap qy								Gǒu zǎo míhóutáo	狗枣猕猴桃
*Sambucus adnata* Wall. ex DC.	qyp ndip		qyp ndi		qyp ndi		qyp ndi		Xuè mǎn cǎo	血满草
*Sambucus williamsii* Hance	syr qyp ndip (si qi nie)	()			ziep gup dda				Jiēgǔ mù	接骨木
*Viburnum betulifolium* Batalin	got bu sup sup (got bbo su su)	()	shot shop						Huà yè jiá mí	桦叶荚蒾
*Amaranthus blitum* L.	sa dip								Āo tóu xiàn	凹头苋
*Celosia argentea* L.	ax ji								Qīng xiāng	青葙
*Chenopodium hybridum* L.	hniet nra (nie zha)	()	vop hly nyix nrat		hnat rra sse		vop hly nyix zhax		Xiǎo lí	小藜
*Allium macrostemon* Bunge	ap hlep wo hmot								Xièbái	薤白
*Allium ovalifolium* Hand.-Mazz.	pa suo								Luǎn yè jiǔ	卵叶韭
*Toxicodendron vernicifluum* (Stokes) F.A. Barkley	jy bbo								Qīshù	漆树
*Angelica sinensis* (Oliv.) Diels	nyie lyt								Dāngguī	当归
*Oenanthe javanica* (Blume) DC.	yy zyx lo bbo				ax lie wop				Shuǐ qín	水芹
*Metaplexis japonica* (Thunb.) Makino	ka ba ji ji								Luó mó	萝藦
*Aralia chinensis* L.	ax pu vop nzi		si vop nzi						Sǒng mù	楤木
*Aristolochia versicolor* S.M.Hwang	map yox (ma yo)	()							Biànsè mǎ dōu líng	变色马兜铃
*Polygonatum cyrtonema* Hua	va bu qy ap lip								Duō huā huángjīng	多花黄精
*Begonia grandis* subsp. *sinensis* (A.DC.) Irmsch.	chur hie tap bbo								Qiūhǎitáng	秋海棠
*Berberis jamesiana* Forrest & W.W.Sm.	va dot		six si		yiep ddit				Chuān diān xiǎo bò	川滇小檗
*Mahonia bealei* (Fortune) Pynaert	va mu gep du (va mu ge du)	()			gep di				Kuò yè shí dà gōngláo	阔叶十大功劳
*Incarvillea diffusa* Royle	vat bbu yox								Liǎngtóu máo	两头毛
*Cynoglossum amabile* Stapf & J.R.Drumm.	mip si (mi si)								Dào tí hú	倒提壶
*Capsella bursa-pastoris* (L.) Medik.	ci zi vap ga								Jì	荠
*Cardamine tangutorum* O.E.Schulz	ot vop		yy yyx ap zhat		it wop		it vop		Zǐhuā suì mǐ jì	紫花碎米荠
*Nasturtium officinale* R.Br.	yy wox								Dòubàn cài	豆瓣菜
*Rorippa dubia* (Pers.) H.Hara	vo pi								Wú bàn hǎn cài	无瓣蔊菜
*Hylocereus undatus* (Haw.) Britton & Rose	ho lop max ma								Liàng tiān chǐ	量天尺
*Opuntia ficus-indica* (L.) Mill.	nyit cy bbu ga								Lí guǒ xiānrénzhǎng	梨果仙人掌
*Codonopsis pilosula* subsp. *tangshen* (Oliv.) D.Y.Hong	va bu sha ggox		wo mu rry nyix		kep sse hep ddu		jix rry hex ddur		Chuān dǎngshēn	川党参
*Leycesteria formosa* Wall.	wa ji								Guǐ chuī xiāo	鬼吹箫
*Arctium lappa* L.	ax jju le bbu (a jju le bbu)								Niúbàng	牛蒡
*Artemisia capillaris* Thunb.	hxix ke qu								Yīn chén hāo	茵陈蒿
*Cirsium shansiense* Petr.	vot mop qu got		nry ke		jjyp kuop		nry ke		Niú kǒu cì	牛口刺
*Eclipta prostrata* (L.) L.	bu mu ce ke								Lǐ cháng	鳢肠
*Kalimeris indica* (L.) Sch.Bip.	qie la, jie nuo								Mǎlán	马兰
*Pseudognaphalium affine* (D.Don) Anderb.	viep vie a shy				jot vit		zhut vit		Shǔ qū cǎo	鼠麴草
*Sonchus oleraceus* (L.) L.	ax jju mit jy								Kǔ jù cài	苦苣菜
*Taraxacum mongolicum* Hand.-Mazz.	pup go yi bbo		vot mop hxop ke						Púgōngyīng	蒲公英
*Cornus kousa* subsp. *chinensis* (Osborn) Q.Y.Xiang	vot mop syp njo		syp nji li bbi		si ji le bbox		syp nji li bbi		Sì zhào huā	四照花
*Trichosanthes kirilowii* Maxim.	guo bbo								Guā lóu	栝楼
*Araiostegia divaricata* var. *formosana* (Hayata) M. Kato	a zhat bat ji								Dà yè gǔ suì bǔ	大叶骨碎补
*Pteridium aquilinum* (L.) Kuhn	ndax bbo								Jué	蕨
*Dioscorea polystachya* Turcz.	ax hxie yiep yot		ax nuo syp hmi		a ge da lie				Shǔyù	薯蓣
*Diospyros lotus* L.	got du ax nuo						nyi bbu		Jūn qiān zi	君迁子
*Elaeagnus pungens* Thunb.	syr huo		syr fi				mu mu cep hlo		Hú tuí zi	胡颓子
*Equisetum giganteum* L.	ry zuo								Bǐ guǎn cǎo	笔管草
*Vaccinium fragile* Franch.	wax ma chu qy		yie ry		yiep ma sry nip				Wūyā guǒ	乌鸦果
*Eucommia ulmoides* Oliv.	dup zhop								Dùzhòng	杜仲
*Quercus schottkyana* Rehder & E.H.Wilson	si ji max ma								Diān qīnggāng	滇青冈
*Helwingia japonica* (Thunb.) F.Dietr.	cy lyr								Sìchuān qīng jiá yè	四川青荚叶
*Iris forrestii* Dykes	mie ci								Yuānwěi	鸢尾
*Mentha canadensis* L.	yo zhet						vot mop qy zot		Bòhé	薄荷
*Perilla frutescens* (L.) Britton	hxie zy mip (mu)		hly hxop		syt map		mup hex ap vop		Zǐ sū	紫苏
*Akebia trifoliata* (Thunb.) Koidz.	yox sse la bbox (yo re la bo)		la bbo la ot				la bbo		Sān yèmù tōng	三叶木通
*Decaisnea insignis* (Griff.) Hook.f. & Thomson	la yot								Māo er shǐ	猫儿屎
*Litsea cubeba* (Lour.) Pers.	mu suo								Shān jī jiāo	山鸡椒
*Litsea pungens* Hemsl.	mux ku								Mù jiāng zi	木姜子
*Pueraria montana* var. *lobata* (Willd.) Sanjappa & Pradeep	ge wop nyip ggu								Gé	葛
*Spatholobus suberectus* Dunn	nyip ggu syr du				jup hxa				Mì huā dòu	密花豆
*Vicia sativa* L.	sha nuo mu re								Jiù huāngyě wāndòu	救荒野豌豆
*Fritillaria cirrhosa* D.Don	yyx syr		ryp sy		ryp sy		ryp sy		Chuān bèi mǔ	川贝母
*Huperzia squarrosa* (G. Forst.) Trevis.	ca na nyip ggu								Cūcāo mǎwěi shān	粗糙马尾杉
*Lycopodium japonicum* Thunb.	shyp so nyip ggu		six mip						Shísōng	石松
*Malva verticillata* L.	ax yie				ap yiep		ap yit		Dōng kuí	冬葵
*Paris polyphylla* Sm.	map bup								Qī yè yīzhī huā	七叶一枝花
*Toona sinensis* (Juss.) M.Roem.	syr wo		six e vot zza		syr yy		six e		Xiāngchūn	香椿
*Ficus pumila* L.	si jie le bi								Bì lì	薜荔
*Ficus tikoua* Bureau	kex six vot six (si ke wu si)		si ke vot six		qy syr jie		si ke le hu		Dìguǒ	地果
*Morus australis* Poir.	ax jji bbu zza								Jī sāng	鸡桑
*Musa basjoo* Siebold & Zucc. ex Iinuma	ba jo								Bājiāo	芭蕉
*Myrica nana* A. Chev.	syp vyt								Yúnnán yángméi	云南杨梅
*Matteuccia struthiopteris* (L.) Tod.	ndax yi (nda yi)		nda o		ndap jjop				Jiáguǒ jué	荚果蕨
*Ophioglossum vulgatum* L.	a mat va hxa								Píng ěr xiǎo cǎo	瓶尔小草
*Bulbophyllum odoratissimum* (Sm.) Lindl. ex Wall.	jux ha (ju ha)								Mì huāshí dòu lán	密花石豆兰
*Gastrodia elata*	bbup shy		yo la bbup shy				yo la bbup shy		Tiānmá	天麻
*Osmunda japonica* Thunb.	lot ni				ndap jjop				Zǐ qí	紫萁
*Oxalis corniculata* L.	a zhat vop ji		a bbu ji ji		a zhat wop ji		ap bbux zax jip		Cù jiāng cǎo	酢浆草
*Plantago major* L.	vot mop ddip bbu								Píng chē qián	平车前
*Fargesia spathacea* Franch.	map mut								Jiànzhú	箭竹
*Imperata cylindrica* (L.) Raeusch.	bbo ry				ryp pu				Báimáo	白茅
*Reynoutria multiflora* (Thunb.) Moldenke	a vu yiep yot		yop mop qie bbuo		yop mop qie bbuo				Héshǒuwū	何首乌
*Lemmaphyllum carnosum* (J. Sm. ex Hook.) C. Presl	sip rux (si ru)								Ròuzhì fúshí jué	肉质伏石蕨
*Pyrrosia lingua* (Thunb.) Farw.	put viep								Shí wéi	石韦
*Lysimachia congestiflora* Hemsl.	re na bbo								Jù huā guòlù huáng	聚花过路黄
*Anemone vitifolia* Buch.-Ham. ex DC.	ax jju sha bbu (a jju sha bbu)		ax bbu xi bbu		ax bbu xie bbur				Yě miánhuā	野棉花
*Hovenia dulcis* Thunb.	a zhat xix si								Běi zhǐ jǔ	北枳椇
*Agrimonia pilosa* Ledeb.	e shy e ma								Lóng yá cǎo	龙芽草
*Crataegus scabrifolia* (Franch.) Rehder	syp bu								Yúnnán shānzhā	云南山楂
*Duchesnea indica* (Jacks.) Focke	bbu shy ddut zza		bbu shy vat zha		bbu shy zza		bbu shy cep hlo		Shé méi	蛇莓
*Fragaria nilgerrensis* Schltdl. ex J.Gay	cep hlo				cep hlep		cep hlo iet zyr		Huáng máo cǎoméi	黄毛草莓
*Potentilla discolor* Bunge	mux sip ap mat (mu si a ma)		cep hlo njip njip		cep hlep a ni ni				Fān bái cǎo	翻白草
*Prunus trichostoma* Koehne	ax ji lu ga		vip nyix						Chuānxī yīngtáo	川西樱桃
*Pyracantha fortuneana* (Maxim.) H.L.Li	va bu zzax jjy		ap njit		ap jjiep				Huǒ jí	火棘
*Pyrus pashia* Buch.-Ham. ex D.Don	syp ndat lat qu				syp ndat lat sse		syp nda		Chuān lí	川梨
*Rosa omeiensis* Rolfe	ma pyt syt pyp (ma pu si pi)		syt pyp				va pyt syt pyp		Éméi qiángwēi	峨眉蔷薇
*Rosa roxburghii* Tratt.	syp hni (si ni)				jut lu shax shax				Dān bàn sāo sī huā	单瓣缫丝花
*Rubus ellipticus* var. *obcordatus* (Franch.) Focke	ax nyie sit sip (ax ni se se)		cep hlo		cep hlep a shy shy		cep hlo		Zāi yāng pào	栽秧泡
*Rubus inopertus* (Focke) Focke	cex lop ap nyi nyix (ce le a ni ni)		shot shop cep hlo						Hóng huā xuán gōuzi	红花悬钩子
*Rubus mesogaeus* Focke	sha shax cep hlo (sha sha ce le)								Xǐ yīn xuán gōuzi	喜阴悬钩子
*Rubus wallichianus* Wight & Arn.	a nyie sit sip								Hóng máo xuán gōuzi	红毛悬钩子
*Houttuynia cordata* Thunb.	zyp vo				zip wo				Jí cài	蕺菜
*Schisandra rubriflora* Rehder & E.H.Wilson	yox sse syp yo (yox sse syr yo)								Hóng huā wǔwèizi	红花五味子
*Smilax stans* Maxim.	ax ga lat qu (a ga la qu)								Qiào bǐng bá qiā	鞘柄菝葜
*Physalis alkekengi* L.	nyit a mu se se								Suān jiāng	酸浆
*Vitis heyneana* Roem. & Schult*.*	vot mop syp hxo		yox zzi sup sup		syp wox bbuo zuo sse				Máo pútáo	毛葡萄
*Hemerocallis citrina* Baroni	pop vie								Huánghuā cài	黄花菜

### The preparation and consumption pattern of WEPs

#### Primary foods

Of the 7 primary consumption patterns of WEPs in Liangshan, 40 species are used as primary foods, and of these, the most commonly consumed parts are tender shoots (18 species) and roots (8 species). Usually, the tender shoots, leaves, and flowers are made into soups (5 species), pickled (5 species), eaten raw as salad greens (2 species), or eaten after boiling in water (21 species) (Table 4). The roots tend to be stewed with pork or chicken (11 species), not only for nourishment, but also for the prevention and treatment of diseases. For example, the roots of *Arctium lappa*, *Cirsium shansiense*, *Codonopsis pilosula* subsp. *tangshen*, and *Ophioglossum vulgatum* are generally stewed with chicken or pork used as a tonic. *Gastrodia elata* is stewed to relieve headaches, *Arctium lappa* is also stewed for detoxification, and *Imperata cylindrical* is stewed to reduce cough.

**Table 4:** The primary consumption patterns of WEPs in Liangshan

Consumption pattern	Mode of consumption	Species
Primary food	Boiled in water	(1) *Allium ovalifolium*, (2) *Amaranthus blitum*, (3) *Aralia chinensis*, (4) *Chenopodium hybridum*, (5) *Fargesia spathacea*, (6) *Hemerocallis citrina*, (7) *Hylocereus undatus*, (8) *Kalimeris indica*, (9) *Matteuccia struthiopteris*, (10) *Oenanthe javanica*, (11) *Opuntia ficus-indica*, (12) *Osmunda japonica*, (13) *Perilla frutescens*, (14) *Plantago major*, (15) *Pteridium aquilinum*, (16) *Smilax stans*, (17) *Sonchus oleraceus*, (18) *Taraxacum mongolicum*, (19) *Toona sinensis,(20) *Toxicodendron vernicifluum*, (21) *Vicia sativa*
	Made into pickles	(1) *Begonia grandis* subsp. *sinensis*, (2) *Nasturtium officinale*, (3) *Oenanthe javanica*, (4) *Rorippa dubia*, (5) *Smilax stans*
	Made into soup	(1) *Capsella bursa-pastoris*, (2) *Cardamine tangutorum, (3) *Helwingia japonica*, (4) *Nasturtium officinale*, (5) *Rorippa dubia*
	Raw as salad greens	(1) *Allium macrostemon, (2) *Houttuynia cordata
	Stewed with pork or chicken	(1) *Angelica sinensis, (2) *Arctium lappa, (3) *Cirsium shansiense, (4) *Codonopsis pilosula subsp. *tangshen*, (5) *Dioscorea polystachya*, (6) *Eucommia ulmoides, (7) *Gastrodia elata, (8) *Imperata cylindrica, (9) *Musa basjoo, (10) *Ophioglossum vulgatum, (11) *Polygonatum cyrtonema
Famine food	Food supplement	(1) *Anemone vitifolia*, (2) *Artemisia capillaris*, (3) *Celosia argentea*, (4) *Pseudognaphalium affine*, (5) *Pyracantha fortuneana*
	Starch extraction	(1) *Pteridium aquilinum*
Snack	Eaten raw	(1) *Actinidia kolomikta*, (2) *Akebia trifoliata*, (3) *Berberis jamesiana*, (4) *Cornus kousa* subsp. *chinensis*, (5) *Crataegus scabrifolia*, (6) *Decaisnea insignis*, (7) *Diospyros lotus*, (8) *Duchesnea indica*, (9) *Elaeagnus pungens*, (10) *Ficus pumila*, (11) *Ficus tikoua*, (12) *Fragaria nilgerrensis*, (13) *Hovenia dulcis*, (14) *Mahonia bealei*, (15) *Morus australis*, (16) *Metaplexis japonica*, (17) Morus austrails, (18) *Myrica nana*, (19) *Musa basjoo*, (20) *Opuntia ficus-indica*, (21) *Oxalis corniculata*, (22) *Physalis alkekengi*, (23) *Prunus trichostoma*, (24) *Pueraria montana* var. *lobata*, (25) *Pyracantha fortuneana*, (26) *Pyrus pashia*, (27) *Rosa omeiensis*, (28) *Rosa roxburghii*, (29) *Rubus ellipticus* var. *obcordatus*, (30) *Rubus inopertus*, (31) *Rubus mesogaeus*, (32) *Rubus wallichianus*, (33)*Sambucus adnata*, (34) *Schisandra rubriflora, (35) *Vaccinium fragile*, (34) *Viburnum betulifolium*, (36) *Vitis heyneana*
	Roasted or cooked	(1) *Dioscorea polystachya*, (2) *Quercus schottkyana*
Spice	Seasoning	(1) *Allium macrostemon*, (2) *Houttuynia cordata*, (3) *Litsea cubeba*, (4) *Litsea pungens*, (5) *Mentha canadensis*, (6) *Perilla frutescens*
	Sour flavor enhancer	(1) *Berberis jamesiana*, (2) *Viburnum betulifolium*, (3) *Oxalis corniculata*
Culinary coagulant	Making cheese	(1) *Reynoutria multiflora*
	Making tofu	(2) *Berberis jamesiana*
Medicine	Eaten raw	(1) *Fritillaria cirrhosa*, (2) *Gastrodia elata*
	External use	(1) *Artemisia capillaris*, (2) *Fritillaria cirrhosa*, (3) *Opuntia ficus-indica*, (4) *Paris polyphylla*
	Medicinal soup	(1) *Leycesteria formosa*, (2) *Malva verticillata*, (3) *Sambucus adnata*, (4) *Sambucus williamsii*
	Medicinal tea	(1) *Agrimonia pilosa*, (2) *Berberis jamesiana*, (3) *Bulbophyllum odoratissimum*, (4) *Cynoglossum amabile*, (5) *Eclipta prostrata*, (6) *Huperzia squarrosa*, (7) *Imperata cylindrica*, (8) *Incarvillea diffusa*, (9) *Iris forrestii*, (10) *Kalimeris indica*, (11) *Lemmaphyllum carnosum*, (12) *Lycopodium japonicum*, (13) *Lysimachia congestiflora*, (14) *Mahonia bealei*, (15) *Mentha canadensis*, (16) *Plantago major*, (17) *Potentilla discolor*, (18) *Pyrrosia lingua*, (19) *Spatholobus suberectus*, (20) *Taraxacum mongolicum*, (21) *Trichosanthes kirilowii*
	Tincture	(1) *Araiostegia divaricata* var. *formosana*, (2) *Aristolochia versicolor*, (3) *Crataegus scabrifolia*, (4) *Eclipta prostrata*, (5) *Equisetum giganteum*, (6) *Gastrodia elata*, (7) *Hovenia dulcis*, (8) *Lysimachia congestiflora*, (9) *Reynoutria multiflora*, (10) *Paris polyphylla*, (11) *Potentilla discolor*
Other use	Hedge	(1) *Pyracantha fortuneana*
	Honey collection	(1) *Pseudognaphalium affine*
	Kindling	(1) *Pseudognaphalium affine*
	Rituals	(1) *Aralia chinensis*, (2) *Artemisia capillaris*, (3) *Sambucus adnata*, (4) *Toxicodendron vernicifluum*
	Silver jewelry polish	(1) *Oxalis corniculata*

*With medicinal effect

**Fig. 2:**
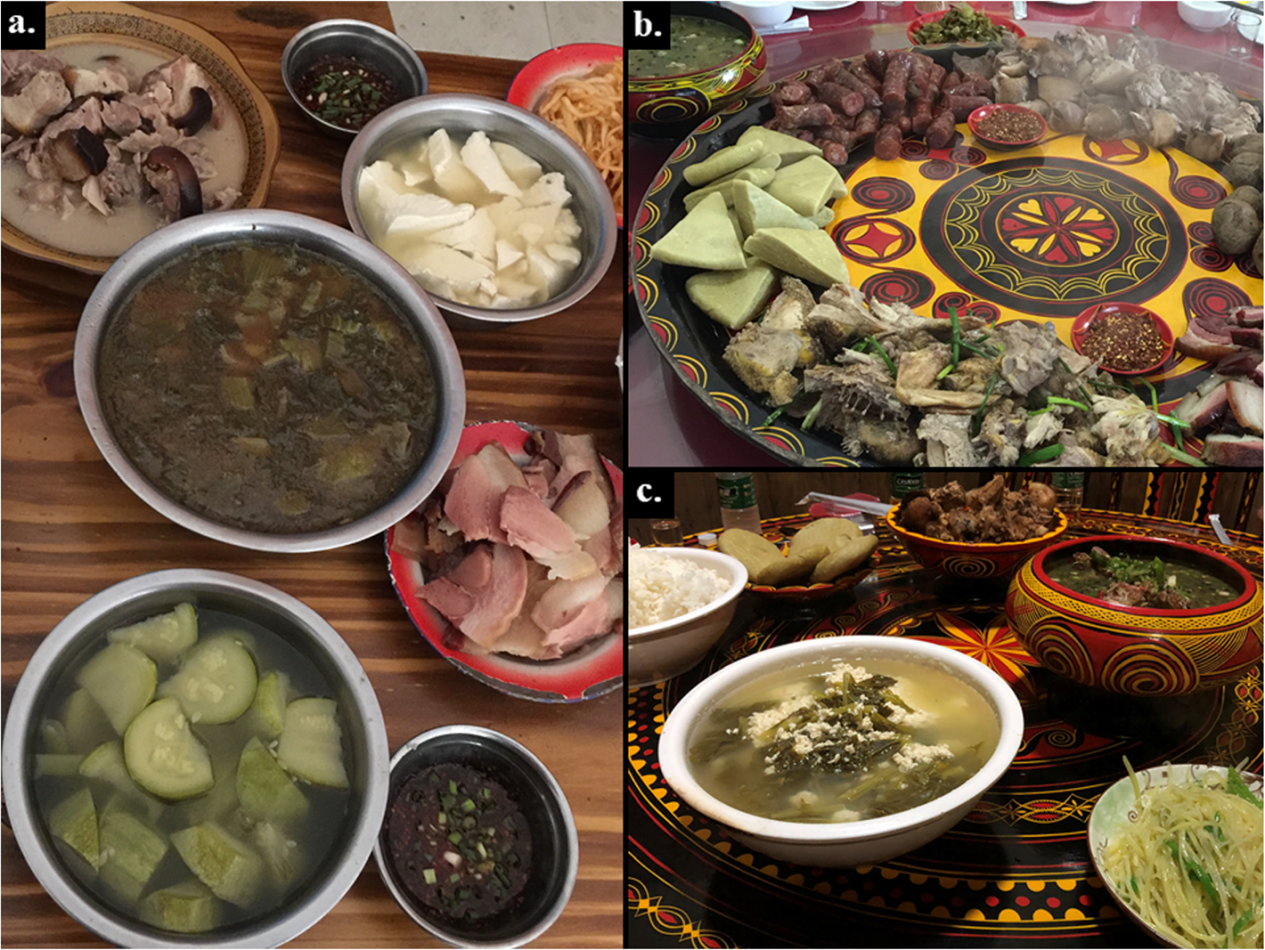
Typical examples of Liangshan Yi cuisine. **a***Suāncài tāng* or pickle soup (center bowl; Zhaojue County). **b** From top left corner clockwise: *Suāncài tāng* (pickle soup), sausage, *tuótuó ròu* (lump pork), boiled potatoes, ham, *tuótuó jī* (lump chicken), and bitter buckwheat cakes (Butuo County). **c** A meal with two types of *Suāncài tāng* (pickle soup), *tuótuó ròu* (lump pork), and bitter buckwheat cakes (Zhaojue County). Photo credits: **a**, **b** JW; **c** BCS

One of the distinctive aspects of the traditional Liangshan Yi cuisine is sour soups, with a bowl of sour soup at almost every meal (Fig. 2). There are five species of WEPs used for making pickled (lacto-fermented) vegetables that are the basis for these soups: *Begonia grandis* subsp. *sinensis*, *Nasturtium officinale*, *Oenanthe javanica*, *Rorippa dubia*, and *Smilax stans*. These are prepared by putting the plant materials in boiling water for 1–2 min, then placing them into a bucket, with the addition of some salt, sealed and left for about half a month. The fermented WEPs become sour in flavor, and they are then stewed with potatoes, beans, and chicken, or made alone into soup. Some of the most famous Yi dishes are *suāncài tāng* (pickle soup) and *suāncài jī tāng* (pickle and chicken soup).

#### Famine foods

Five species of WEPs are eaten as food supplements in times of famine, including the seeds with pappus of *Anemone vitifolia*, tender shoots of *Artemisia capillaris*, seeds of *Celosia argentea*, flowers of *Pseudognaphalium affine*, and the ground-up fruits of *Pyracantha fortuneana*. Each of these can be mixed with buckwheat flour or cornmeal, which increases the volume of cakes for meals and increases their nutritive value as well. A single WEP is used to extract starch during famines. The roots of *Pteridium aquilinum* are crushed in water, and the starch is obtained through sedimentation and filtration. This starch is then used for making cakes after it is dried.

#### Snacks

Most of the WEPs eaten as snacks (38 species) are wild fruits, such as *Akebia trifoliata*, *Cornus kousa* subsp. *chinensis*, *Elaeagnus pungens*, *Fragaria nilgerrensis*, *Pyracantha fortuneana*, and *Rubus* sp. These are often consumed by Yi children when they are herding livestock. The Yi shepherd children also roast bulbils of *Dioscorea polystachya* and the seeds of *Quercus schottkyana* for snacks in the wild. The fresh roots of *Pueraria montana* var. *lobata*, which are also sold in the market for about ¥20 per kilogram, are cut into thin slices and eaten as snacks.

#### Spices

**Fig. 3:**
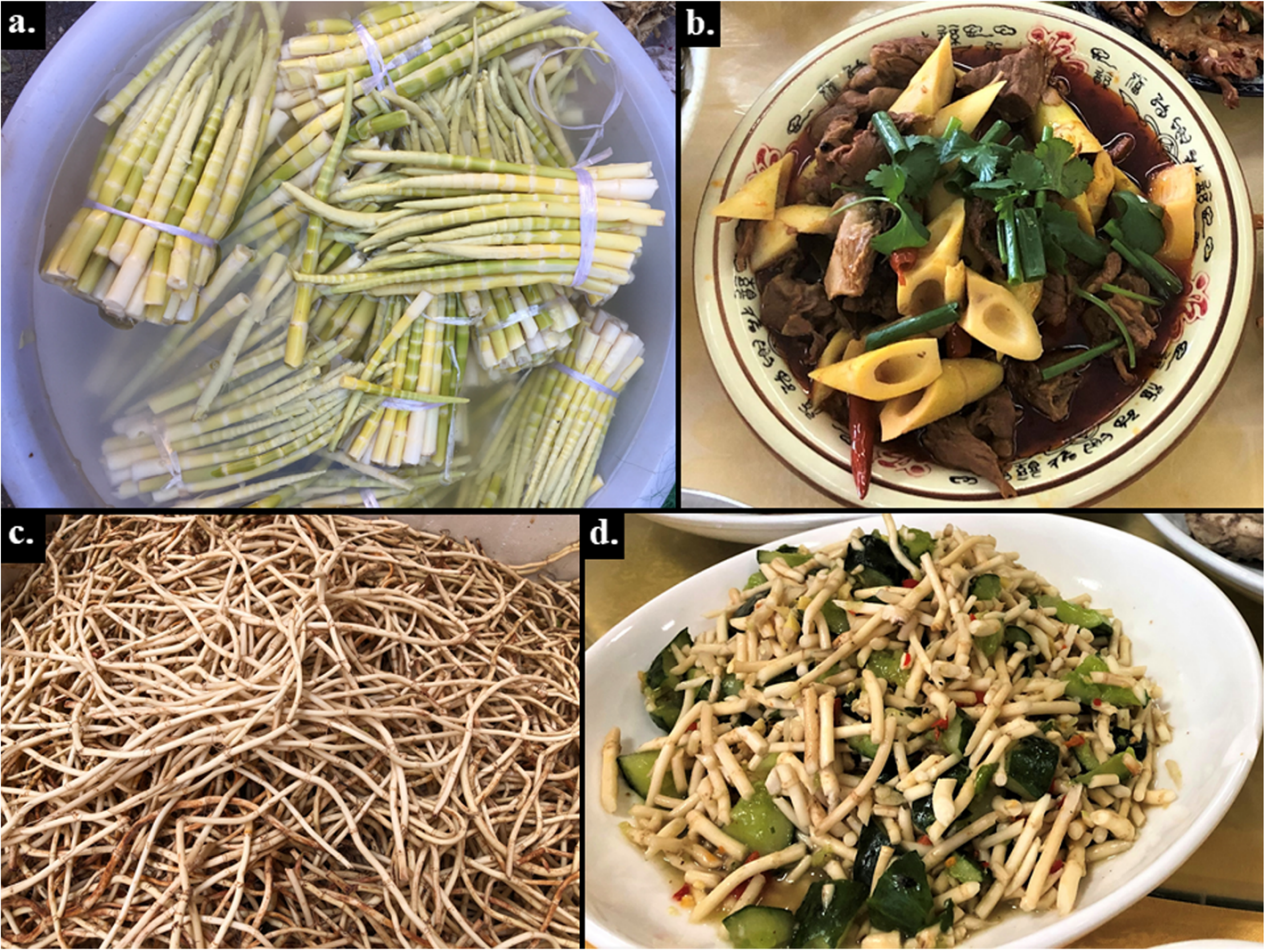
Before and after. **a**, **b** The young shoots of bamboo (*Fargesia spathacea*) are used in many dishes, including bamboo shoots and braised beef (Xichang City). **c**, **d** The chopped roots of *Houttuynia cordata* are used in many Liangshan Yi dishes, including in this cold salad (Puge County). Photo credits: **a** JW; **b**–**d** BCS

There are nine species of WEPs used as spices, among which six species are seasonings. These include *Litsea pungens* and *L. cubeba* widely used in the local cuisine, being essential seasonings in such distinctive Yi dishes as *tuótuó ròu* (lump pork), *tuó tuó jī* (lump chicken), and *suāncài tāng* (pickle soup; Fig. 2). They are prepared by crushing the fresh ripe fruits of *L. pungens* and *L. cubeba* or by scraping the roots with a knife to form a powder. Similarly, wild onion (*Allium macrostemon*) and mint (*Mentha canadensis*) can be added to beef and/or mutton soup to enhance their flavor profiles by masking the strong meaty taste. The seeds of *Perilla frutescens* are fried then ground into powder and mixed with the flour of *Fagopyrum tataricum* to season cakes. The chopped roots of *Houttuynia cordata* mixed with soy sauce, vinegar, salt, and chili powder are used as a seasoning sauce (Fig. 3). The Liangshan Yi utilize three WEPs as flavor enhancers to increase the sourness of the soups beyond what the pickles provide, including the fruits of *Berberis jamesiana* and *Viburnum betulifolium*, as well as the leaves of *Oxalis corniculata*.

#### Culinary coagulants

There are two species used as culinary coagulants. The leaves of *Reynoutria multiflora* are crushed and put in goat’s milk. The milk then solidifies, and its smell is effectively masked. It can be eaten directly or made into cheese that can be preserved longer and is considered more delicious than fresh milk. To prepare the cheese, fern leaves are placed on the top and bottom of the milk block, then most of the water is squeezed out with a spoon. Similarly, the Yi people put the juice of *Berberis jamesiana* into soy milk, and the soy milk solidifies and becomes tofu.

#### Medicinal edible plants

According to our survey, the Liangshan Yi use at least 49 species of medicinal edible plants. Of these, several of them have multiple medical applications, with 21 species used to make medicinal tea, 11 species for tinctures, 4 species each for medicinal soups or used externally, and 2 species consumed raw as medicine. Overall, including 13 non-overlapping species from the 15 used as primary foods (the “Primary foods” section) or snacks (the “Snack” section) with secondary medicinal effects, the Liangshan Yi use WEPs to treat 27 ailments (Table 5), including cough, diarrhea, injury, rheumatism, and headaches. There are 10 species used to treat cough, 8 each for treating diarrhea and for tonification, 5 to treat injuries, and 4 each for treating rheumatism and headache.

**Table 5:** Symptoms treated by medicinal WEPs among the Liangshan Yi

Number	Symptom	Species	Medical plants (no. of informants)
1	Bone fracture	2	*Sambucus adnata* (142), *Sambucus williamsii* (207)
2	Cold	1	*Equisetum giganteum* (56)
3	Cough	10	*Bulbophyllum odoratissimum* (100), *Crataegus scabrifolia* (45), *Eclipta prostrata* (89), *Fritillaria cirrhosa* (258), *Imperata cylindrica* (112), *Iris forrestii* (178), *Lemmaphyllum carnosum* (97), *Plantago major* (105), *Taraxacum mongolicum* (236), *Trichosanthes kirilowii* (114)
4	Delivery	1	*Malva verticillata* (270)
5	Detoxification	1	*Arctium lappa* (78)
6	Diarrhea	8	*Agrimonia pilosa* (179), *Berberis jamesiana* (126), *Eclipta prostrata* (153), *Kalimeris indica* (34), *Mahonia bealei* (53), *Plantago major* (56), *Potentilla discolor* (201), *Toona sinensis* (23)
7	Dyspepsia	1	*Houttuynia cordata* (34)
8	Enteritis	1	*Cynoglossum amabile* (198)
9	Gallstone	1	Codonopsis pilosula subsp. tangshen (15), Lysimachia congestiflora (220), Pyrrosia lingua (138)
10	Gastroenteritis	1	*Aristolochia versicolor* (12)
11	Gastropathy	3	*Allium macrostemon* (27), *Huperzia squarrosa* (35), *Potentilla discolor* (137)
12	Headache	4	*Aristolochia versicolor* (38), *Equisetum giganteum* (78), *Gastrodia elata* (134), *Reynoutria multiflora* (159)
13	Heart disease	2	*Musa basjoo* (109), *Spatholobus suberectus* (89)
14	Hemorrhoid	1	*Cynoglossum amabile* (107)
15	Hepatitis	1	*Incarvillea diffusa* (214)
16	Hypertension	2	*Araiostegia divaricata* var. *formosana* (67), *Cardamine tangutorum* (56)
17	Hyperthermia	1	*Mentha canadensis* (43)
18	Injury	5	*Aristolochia versicolor* (52), *Artemisia capillaris* (156), *Fritillaria cirrhosa* (189), *Lemmaphyllum carnosum* (34), *Paris polyphylla* (253)
19	Measles	1	*Leycesteria formosa* (132)
20	Muscle pain	1	*Paris polyphylla* (121)
21	Nephropathy	2	*Eucommia ulmoides* (89), *Cirsium shansiense* (62)
22	Nosebleed	1	*Imperata cylindrica* (45)
23	Pneumonia	1	*Eclipta prostrata* (54)
24	Rheumatism	4	*Allium macrostemon* (27), *Huperzia squarrosa* (78), *Lycopodium japonicum* (95), *Sambucus adnata* (53)
25	Stomachache	1	*Equisetum giganteum* (74)
26	Tonification	8	*Angelica sinensis* (135), *Arctium lappa* (93), *Cirsium shansiense* (126), *Codonopsis pilosula* subsp. *tangshen* (56), *Hovenia dulcis* (75), *Ophioglossum vulgatum* (108), *Polygonatum cyrtonema* (167), Schisandra rubriflora (75)
27	Tonsillitis	1	*Opuntia ficus-indica* (21)

The WEPs used as medicinal teas are prepared by putting the plant materials into boiling water for about 5–10 min. For example, the aboveground parts of *Agrimonia pilosa*, the whole plant of *Potentilla discolor*, and the roots of *Mahonia bealei* and *Berberis jamesiana* are used to cure diarrhea; the roots of *Imperata cylindrica* are used to stop nosebleeds and suppress coughing; and the flowers of *Trichosanthes kirilowii* are also used to treat cough. The aboveground portion of *Incarvillea diffusa* prepared as a tea and mixed with honey and rice wine can treat hepatitis. These teas are usually consumed when someone shows symptoms, drinking approximately 500 ml at a time, 3–5 times a day, until the illness is relieved or cured.

Tinctures are made by putting the plant materials in liquor (ethanol concentration of about 50–65%) and waiting at least half a month before drinking. For treatment, about 50–100 ml is consumed two to three times a day until symptoms subside. For example, *Paris polyphylla* tinctures are used to cure injuries (e.g., bruises caused by a fall or sprains), *Potentilla discolor* tinctures are used to treat diarrhea and gastropathy, *Gastrodia elata* tinctures are used to relieve headaches, and *Lysimachia congestiflora* tinctures are used to treat gallstones.

To make medicinal soups, the plant materials are cooked for a while in a fried egg soup, then the patient consumes the soup. For example, the egg soup of *Malva verticillata* can be used to aid childbirth, the egg soups of *Sambucus adnata* and *S. williamsii* can help heal bone fractures, and the egg soup of *Leycesteria formosa* can cure measles.

Of the two species of WEPs eaten raw as medicine, *Fritillaria cirrhosa* bulbs are collected, sun dried, and stored. The bulbs are then crushed into a powder and ingested orally to treat cough. The powder of dried *Gastrodia elata* rhizomes can be eaten directly to relieve headache.

For the plants used externally, the powder of *Fritillaria cirrhosa* and the ground pulp of *Paris polyphylla* or *Artemisia capillaris* can be applied directly to trauma wounds to quickly stop the bleeding and help wounds heal more rapidly. Some Yi cut the prickly pear cactus’s epidermis (*Opuntia ficus-indica*) and put it on the cheek to treat tonsillitis. It is, however, important to note there are many other medicinal plants used externally by the Liangshan Yi that are not recorded here because they are not WEPs.

#### Other uses

We found that seven species of WEPs also had other non-eating uses. For example, four species are used as ritual plants. *Artemisia capillaris* is used in cleansing rituals. Heated stones are placed in a container with *A. capillaris* and water, causing a white steam to arise filled with the plant’s aromatic oil. The person or thing passing through the steam is considered ceremonially clean. The branches of *Aralia chinensis* are used in the ritual of installing ancestral spirits (i.e., assisting the souls of deceased relatives to reach the spiritual realm). The branches of both *Toxicodendron vernicifluum* and *Sambucus adnata* are used in exorcism rituals.

**Fig. 4:**
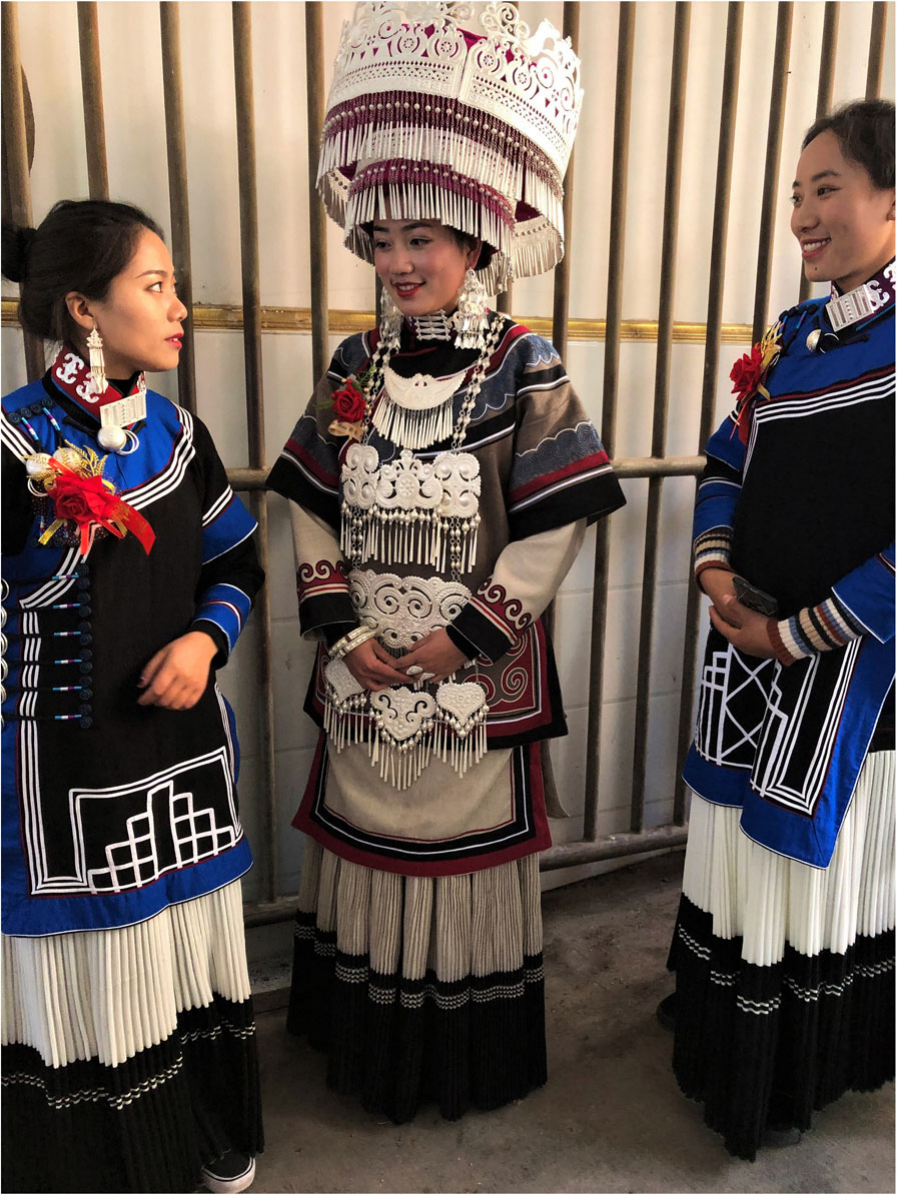
A young bride on her wedding day (Puge County). The bride (center) and bridesmaids are donning traditional silver Yi jewelry and other handicrafts; aboveground portions of *Oxalis corniculata* are traditionally used to de-tarnish and polish the silver attire. Fern fiddleheads are depicted on both the bride’s silver filigree and embroidered dress (e.g., sleeves and waist). Photo credit: BCS

**Fig. 5:**
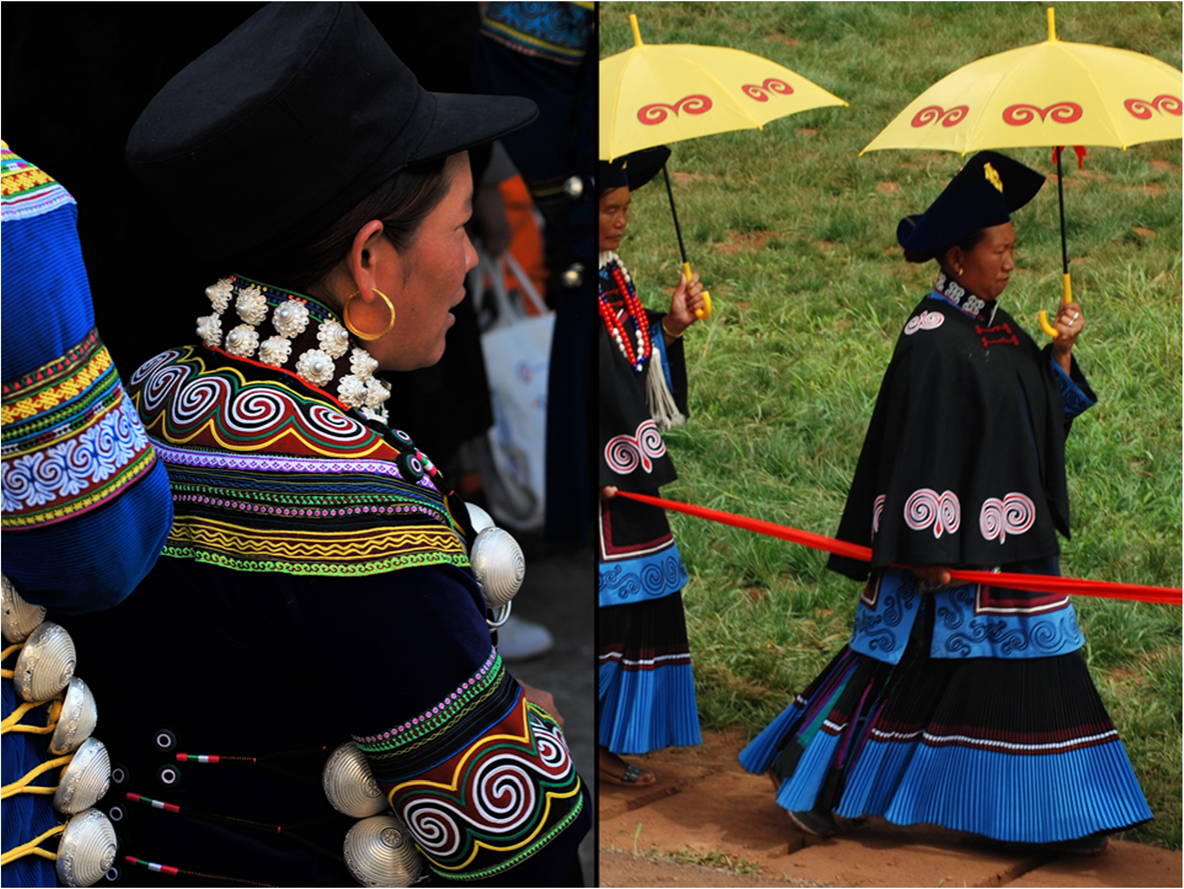
Fern fiddleheads, representing fertility and abundance, are frequently depicted on Liangshan Yi women’s traditional attire. Puge County (left) and Xide County (right). Photo credits: BCS

Liangshan Yi collect and dry *Pseudognaphalium affine* as a type of kindling. Some elder Yi often carry steel, flint, and kindling in their pouches. When they want to smoke, they strike the steel against the flint to generate a spark, setting the prepared kindling on fire to ignite the pipe. Secondly, when Yi hunters find a wild beehive, in order to collect honey, the dried vegetation of *P. affine* is ignited under the hive, and the resulting smoke dispels the bees and/or stuns them, so that the hunter can easily obtain the honey. Yi women use *Oxalis corniculata* to scrub, de-tarnish, and polish silver jewelry (earrings, rings, bracelets, etc.), which are important items in Yi traditional attire (Figs. 4 and 5). *Pyracantha fortuneana* is a very common hedge plant and is planted around the yard perimeters in many Yi villages.

### Months of collection

**Fig. 6:**
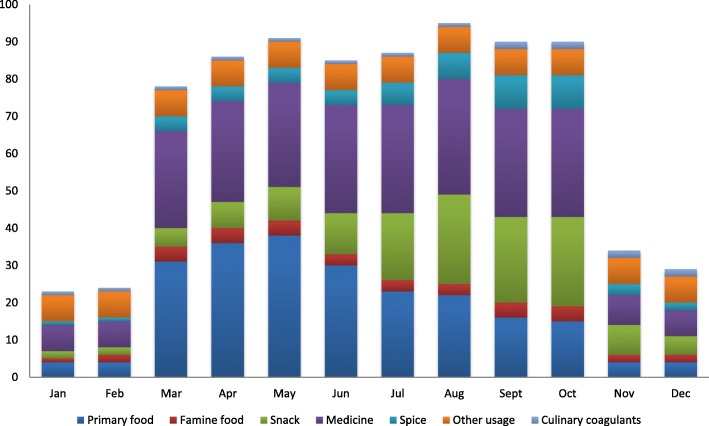
Months of collection for WEPs in Liangshan

The Liangshan Yi mainly collect WEPs from March to October (Fig. 6). The greatest number of species can be collected in August (95 species), followed by May (91), September and October (90 each), July (87), April (86), June (85), and March (78). Due to inclement weather and frost, the number of WEPs collected during late autumn and winter is much fewer. For instance, only 34 species are collected in November, 29 species in December, 24 species in February, and only 23 species in January.

Most of the WEPs used for food are collected from March to June, with the total number gradually declining through October, with only four species collected year-round. Wild fruits consumed as snacks as well as spices are most often collected from July to October. Medicinal plants are mainly collected from March to October. For culinary coagulants, the leaves of *Reynoutria multiflora* are collected all year-round, but the fruits of *Berberis jamesiana* are only collected when mature (September to December). All seven WEPs in the “other uses” category are collected year-round.

### Commercial valuation

Many Liangshan Yi collect certain species of WEPs to sell at the market to augment their household incomes. We found 35 species of WEPs sold in the markets we surveyed, with 8 species sold as food, 18 species as medicinal herbs, 6 species as wild fruits (including the roots of *Pueraria montana* var. *lobata* eaten as a snack), and 3 species as spices (Table 2).

The prices of medicinal WEPs (¥40–2000 RMB/kg; note: $1 USD = ¥6.8 RMB) were much higher than the price of foods and fruits (¥5–20 RMB/kg; Table 2). *Fritillaria cirrhosa*, *Paris polyphylla*, *Gastrodia elata*, *Reynoutria multiflora*, *Angelica sinensis*, *Spatholobus suberectus*, *Huperzia squarrosa*, and *Ophioglossum vulgatum* were targets of commercial acquisition, so their prices were particularly high. For example, dry *Fritillaria cirrhosa* sold for ¥2000 RMB/kg and *Paris polyphylla* sold for ¥600 RMB/kg. Fresh *Gastrodia elata* sold for approximately ¥100 RMB/kg, while the dried form sold for ¥500 RMB/kg. In contrast, because of their ample supply and wide distribution, the price of *Taraxacum mongolicum* and *Plantago major* was much lower (¥10 RMB/kg).

*Fritillaria cirrhosa* and *Paris polyphylla* are important raw materials for many traditional Chinese medicine (TCM) preparations. *F. cirrhosa* is a key herb used to make a popular cough syrup called *Chuān bèi pípá gāo* (川贝枇杷膏), which is used by the Chinese diaspora worldwide, including in East Asia, Europe, and North America. *F. cirrhosa* is also one of the most important ingredients in a couple famous Chinese proprietary medicines, including *Yúnnán Báiyào* (云南白药), used for treating injuries and stopping bleeding, and *Gōng Xuè Níng* (宫血宁), used for curing excessive menstruation. It is well-documented that *P. polyphylla* has near-miraculous medicinal properties to cure wounds. External application of the powdered roots can rapidly stop bleeding, reduce inflammation, and even treat venomous snake bites [53–56].

**Fig. 7:**
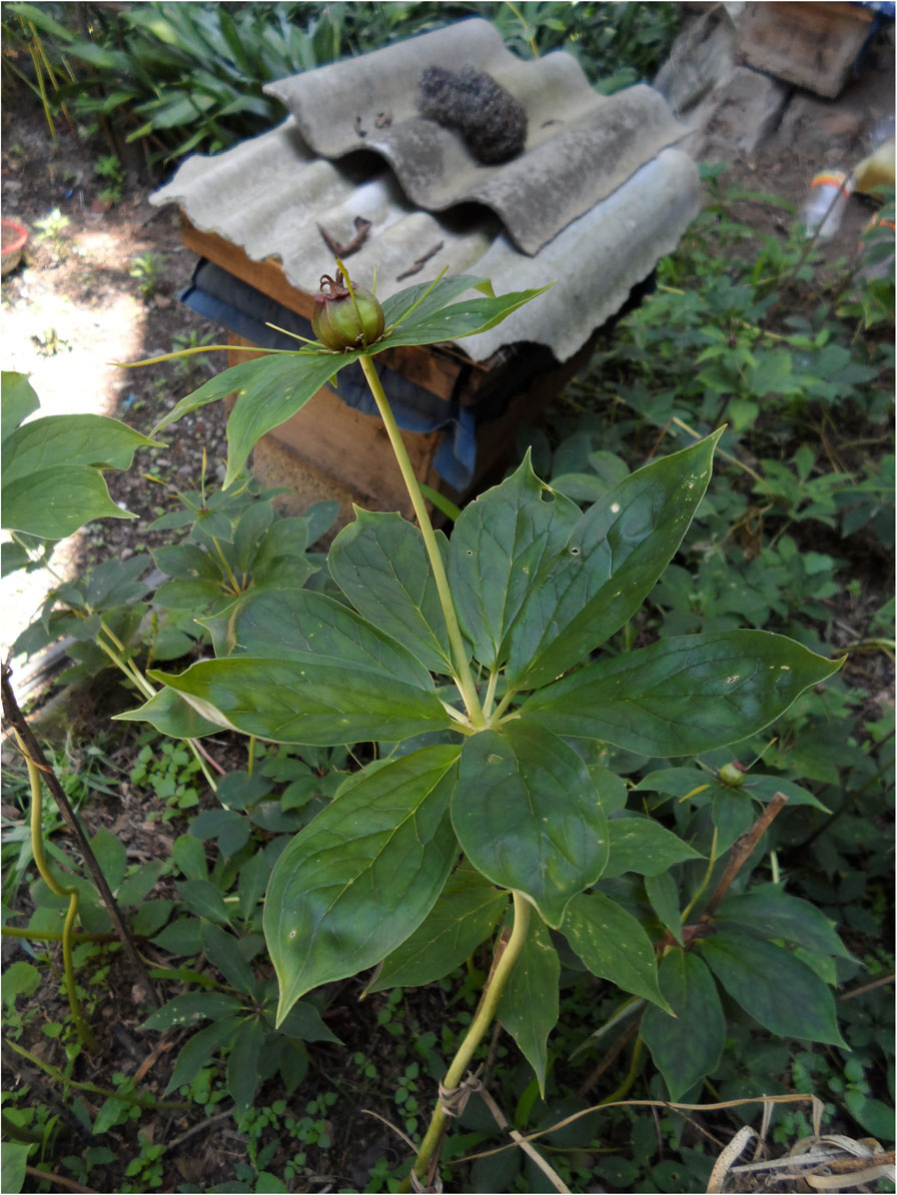
Young plants of *Paris polyphylla* are grown in the courtyards of homes throughout Liangshan, here in Puge County. Photo credit: BCS

Since the 1980s, many pharmaceutical companies began establishing local branches in Liangshan to acquire *Paris polyphylla*. Consequently, interviewees reported that the wild resources of this plant have been so greatly deplenished that it is now very difficult to collect them for local medical needs. Therefore, many locals in Liangshan have begun purchasing wild seedlings of *P. polyphylla* to plant in their courtyards for convenient access when someone is wounded (Fig. 7). They will also sell them after they have grown to maturity. During one of our field surveys in Liangshan (Meigu County, April 2017), we met a local team whose sole aim was to collect wild *P. polyphylla* seedlings. They said that about 2000 individuals could be collected each day, and each plant could be sold to villagers for about ¥0.2 RMB.

### Use value

The five species with the highest use values (UVs) were *Berberis jamesiana* (1.92), *Pyracantha fortuneana* (1.87), *Artemisia capillaris* (1.44), *Pteridium aquilinum* (1.35), and *Houttuynia cordata* (1.26). The species with the lowest UVs were *Lycopodium japonicum* (0.24), *Spatholobus suberectus* (0.22), *Huperzia squarrosa* (0.20), *Araiostegia divaricata* var. *formosana* (0.17), and *Aristolochia versicolor* (0.13) (Tables 2 and 6).

**Table 6:** WEPs with the highest and lowest use values (UVs)

	Family	Scientific name	Usage 1 (frequency)	Usage 2 (frequency)	Usage 3 (frequency)	Usage 4 (frequency)	ΣUs	UVs
Species with the highest UVs	Berberidaceae	*Berberis jamesiana* Forrest & W.W.Sm.	Snack (314)	Spice (209)	Medicine (126)	Culinary coagulant (112)	761	1.92
	Rosaceae	*Pyracantha fortuneana* (Maxim.) H.L.Li	Snack (379)	Other use (196)	Famine food (166)		741	1.87
	Compositae	*Artemisia capillaris* Thunb.	Other use (346)	Medicine (156)	Famine food (67)		569	1.44
	Dennstaedtiaceae	*Pteridium aquilinum* (L.) Kuhn	Primary food (379)	Famine food (157)			536	1.35
	Saururaceae	*Houttuynia cordata* Thunb.	Primary food (357)	Spice (109)	Medicine (34)		500	1.26
	Oxalidaceae	*Oxalis corniculata* L.	Snack (250)	Spice (198)	Other use (13)		461	1.16
	Compositae	*Cirsium shansiense Petr.*	Primary food (326)	Medicine (126)			452	1.14
	Lamiaceae	*Perilla frutescens* (L.) Britton	Spice (289)	Primary food (149)			438	1.11
	Orchidaceae	*Gastrodia elata* Blume	Medicine (296)	Primary food (134)			430	1.09
	Adoxaceae	*Sambucus adnata Wall. ex DC.*	Snack (235)	Medicine (142)	Other use (45)		422	1.07
Species with the lowest UVs	Helwingiaceae	*Helwingia japonica* (Thunb.) F.Dietr.	Primary food (105)				105	0.27
	Equisetaceae	*Equisetum giganteum* L.	Medicine (102)				102	0.26
	Orchidaceae	*Bulbophyllum odoratissimum* (Sm.) Lindl. ex Wall.	Medicine (100)				100	0.25
	Amaranthaceae	*Celosia argentea* L.	Famine Food (99)				99	0.25
	Cactaceae	*Hylocereus undatus* (Haw.) Britton & Rose	Primary food (98)				98	0.25
	Lycopodiaceae	*Lycopodium japonicum Thunb.*	Medicine (95)				95	0.24
	Leguminosae	*Spatholobus suberectus* Dunn	Medicine (89)				89	0.22
	Lycopodiaceae	*Huperzia squarrosa* (G. Forst.) Trevis.	Medicine (78)				78	0.20
	Davalliaceae	*Araiostegia divaricata* var. *formosana* (Hayata) M. Kato	Medicine (67)				67	0.17
	Aristolochiaceae	*Aristolochia versicolor* S.M.Hwang	Medicine (52)				52	0.13

## Discussion

### Yi Culture of WEPs in Liangshan

Although the Liangshan Yi people have their own traditional written language, historically, it was not widely learned by the general public. Instead, it was primarily used only by their traditional ritual specialists, the *bimox*. With the broader Yi population being largely illiterate throughout much of Liangshan’s history, their cultural knowledge and traditional customs were instead transmitted through oral communication techniques [57], including knowledge of WEPs. One way this information was organized for easy memory and transmission was through traditional Yi cultural sayings. These sayings often included information about the WEPs’ flavors, proper collection times, and medical uses. For example, one traditional saying about the good flavor of soup made from *Cardamine tangutorum* says “I should share the vegetable of *Cardamine tangutorum* with my mom but not the soup” (, ). Another saying states, “Eating *Toona sinensis* prevents diarrhea if collected before the cuckoo chirps in spring” (). Realizing that *Houttuynia cordata* has the effect of promoting rapid digestion, a traditional Yi saying warns not to eat it during times of famine. It states, “a satiated people will be hungry soon after eating *Houttuynia cordata*, but hungry people never try it because they will starve” (, ).

### Climate, lacto-fermentation, and collection season

In contrast to the Han people, who primarily settled in river valleys, most Yi people live in mountainous areas in Liangshan, with limited arable land, colder weather, and longer winters, so this has greatly influenced their cultural traditions, preferred WEPs, and associated knowledge. Since there is a shorter time period to cultivate crops and vegetables, to ensure year-round food supplies, the Liangshan Yi preserve vegetables through lacto-fermentation techniques by making pickles.

Lacto-fermentation is a food preservation technique shared by many other people groups around the world [58, 59], as well as elsewhere in China, such as Tibetans in Gansu Province [23], who live in northern latitude and high altitude areas with long winters. The Liangshan Yi believe these pickles are not only appetizing but aid their digestion, which is a well-documented benefit of eating lacto-fermented foods as probiotics [58, 59]. These cultural beliefs are also similar to those of the people living in the eastern part of Gilan Province (North Iran), who use pickled sour orange fruits to fortify their stomachs [60]. The overall nutritive value of lacto-fermented foods and drinks is also recognized by diverse people groups across Eastern Europe, Turkey, and the Caucasus [58].

In addition, Liangshan Yi cuisine is heavily soup-based, because the staple food is dense, high-carbohydrate buckwheat cakes, which require soup to help swallow (Fig. 2). Consequently, a traditional Yi proverb states, “Soup can nourish people for seven days, but meat only for three days” (), indicating their preference for soup [61]. Nevertheless, the pickle soup, a quintessential component of the Liangshan Yi people’s traditional diet finds a cultural parallel with the Eastern European (e.g., Poland, Lithuania, and Belarus) tradition of preparing sour pickle soups from the lacto-fermented shoots and leaves of hogweed (*Heracleum sphondylium*) [58].

We found that the collection dates for most WEPs are concentrated between March and October. The collection of tender shoots, leaves, and aboveground plant parts for food most often occur between March and June, but, as we predicted, the collection of wild edible fruits (food, snack, and spice categories) tend to be collected later in the growing season (summer and autumn). Due to the seasonal ontogeny in high-elevation Liangshan, these collection seasons are similar to those recorded elsewhere in Northern Hemisphere temperate [22] and high-elevation subtropical regions [4, 5]. However, the collection of famine foods, culinary coagulants, and the most valuable medicinal plants, driven more by the interests of necessity, weather, and economics, were less closely related to the growth and reproduction characteristics of the WEPs. Similarly, the collection of all seven “other use” WEPs (including all four ritual plants) is not affected by season, because the parts collected are mostly branches.

### WEP diversity, use values, and cultural significance

In keeping with our expectations, the families Rosaceae (14 species) and Compositae (8 species) had the highest representation of species in this study, but Lamiaceae was only represented by 2 species (Table 2). The greater number of collected WEPs from these families is similar to the patterns documented elsewhere [5, 22, 23, 62], but unlike those studies, other common families (e.g., Asparagaceae, Caprifoliaceae, Liliaceae, and Polygonaceae) were each represented by a single species in our study. This suggests that the relative *abundance* of a family within a particular area is not reason enough for its species to be collected for food or medicine. In this sense, although we did not specifically test the theory of non-random plant selection [17, 26], our data lends further support for this widely tested theory. For example, if the selection/collection of WEPs were truly random or “placebo” (rather than based on many years of experience and local knowledge of the flora), every common/abundant family would be expected to have greater representation among the utilized WEPs.

We might also expect that the common families would have more species with greater UVs, but, instead, we found that species from 9 different families had the top 10 highest UVs (Table 6). Although all 3 families (Compositae, Lamiaceae, and Rosaceae) had species within the top 10 highest UVs, only Compositae had more than 1 (#3 and #6). However, this may be due to the particularly high species diversity in Liangshan, in that despite stiff competition, these species have proven over time to be the most versatile [26, 63]. Nevertheless, when looking at plant UVs, the relative cultural importance of the collective taxa from each family is better appreciated. For example, of the top 20 UVs (Table 2), the proportion of taxa from the 3 families increases: Compositae (3/8 taxa; 37.5%), Lamiaceae (2/2 taxa; 100%), and Rosaceae (6/14; 42.9%), and of the top 30 UVs: Compositae (4/8 taxa; 50.0%), Lamiaceae (2/2 taxa; 100%), and Rosaceae (9/14; 64.3%). So, according to the UVs, a majority of the collected WEPs from all 3 families are among the most culturally important species, and only 2 species (both Compositae) fall within the bottom 30 UVs (#79 and #86; Table 2).

As Gaoue et al. [26] point out, plant use values are essential measures of plant *versatility*. Thus, not only are the high-UV WEPs widely distributed with large populations throughout Liangshan, the Yi people have found these particular species to be very useful in their daily lives. For example, many Yi people since childhood have consumed the fruit of *Berberis jamesiana* (highest UV) and *Pyracantha fortuneana* (UV #2) raw as snacks between meals while collecting firewood or herding. Most adults also know that the juice of *B. jamesiana* can be used as a culinary coagulant to make tofu or as a flavor enhancer (spice) to increase the sourness of pickle soups. Similarly, the dense growth and thorns of *P. fortuneana*, beautiful white flowers, edible red fruit, and long fruiting period make it commonly planted as a hedge.

Nevertheless, historical events and outside influences may have amplified the relative importance of some of these plants. For example, in the 1950s, the National Pharmaceutical Company began commercial acquisition in Liangshan of the root bark of *Berberis jamesiana* and related species (Berberidaceae) to extract and refine the bioactive compound (berberine), which is medically valuable for treating diarrhea and other ailments. The large volume of acquisitions of *B. jamesiana* root bark for extraction of berberine deeply affected the perception (including the local name) of this plant by many Liangshan Yi [28]. This may contribute to why it has the highest UV in our survey. Similarly, *Pyracantha fortuneana* is an important famine food remembered by elderly Yi people who had experienced severe famine years. The fruit was ground into pulp and mixed into Tartary buckwheat (*Fagopyrum tataricum*) flour or cornmeal to make cakes, augmenting the nutrition of the flours and increasing their volumes.

Our results also demonstrate large differences between the economic/commercial valuation and cultural evaluation (UVs) for particular species (Tables 2 and 6). For example, although we found that medicinal WEPs sell at the market for much higher prices than those used for other purposes, of the three most economically valuable medicinal WEPs, only the use value of *Gastrodia elata* (¥100 RMB/kg wet; ¥500 RMB/kg dry) is in the top ten (UV #9). In contrast, *Fritillaria cirrhosa* (¥2000 RMB/kg) and *Paris polyphylla* (¥600 RMB/kg) were more expensive, but they had much lower cultural significance overall (UV #44 and #46, respectively). However, both of these species are *only* used for medicine, while *Gastrodia elata* is also an important food plant widely sold in the markets. Incidentally, because of children’s good eyesight and flexible bodies, they more easily fit in and around bushes. Thus, during the flowering period of this orchid (when it briefly appears above ground), many Yi children are encouraged to participate in collection activities, so children, in particular, tend to be very knowledgeable about this species.

This demonstrates the need for longitudinal studies to measure plant use values within the same cultural context over time (across differing demographic variables) in order to more robustly test ethnobotanical theories. Cultures are dynamic and resilient, constantly adapting to changing conditions, including the introduction of new species and decline of formerly common species [26]. Unlike the *cultural keystone species theory*, which is hard to quantify beyond cultural perceptions of foundationally important species [26, 64, 65], plant use values do not seek to measure *absolute* importance of particular species within a culture, but instead measure their *relative* cultural importance at a given moment (or “snapshot”) in time [26, 66]. Thus, the rank order of UVs should be interpreted with this in mind.

For example, we found that the tender shoots of *Pteridium aquilinum* (UV #4) are widely eaten by the Liangshan Yi as vegetables, but its roots are also used as a famine food. This species is representative of an important cultural reverence more broadly applied by the Yi people to multiple species of ferns, including *Matteuccia struthiopteris* (UV #24). For thousands of years, ferns like these have been an important source of regular nourishment, medicine, and famine food for the Liangshan Yi. The ancient Yi scriptures *Zuò zhāi xiàn yào gōng shēng jīng* (作斋献药供牲经) describe the fern fiddleheads as representative of abundant and prosperous descendants. Therefore, fiddleheads are common decorative motifs on the Liangshan Yi’s clothing, textiles, and other material culture items (Figs. 4 and 5), which highlight their cultural veneration and gratitude to ferns for providing food in times of famine. At the same time, these fern motifs are expressions of hope that their children will also flourish like the ferns [67].

### Edible medicinal plants and conservation

As we hypothesized, many of the WEPs primarily consumed by the Liangshan Yi as *food* also have *medicinal* effects. Some of these are intentionally ingested for their healthful effects as part of an overall “healthy diet.” For example, the rhizomes of *Gastrodia elata* (UV #9), whole plant of *Houttuynia cordata* (UV #5), and roots of *Cirsium shansiense* (UV #7) are eaten as vegetables, understanding their medicinal effects. This further supports the argument put forward by various authors that there is no clear distinction between the concepts of *food* and *medicine* in many cultures [16, 17, 19]. This is also similar to the documented use of medicinal edible plants (e.g., ginger, buckwheat, and bitter melon) as dietary staples among the Yi people of Guizhou Province, particularly in their ancient medical text *Qǐ gǔ shǔ* (启谷署) [68]. In addition, the Yi that live in Xishuangbanna (Lancang River Basin), in southern Yunnan Province, also have the custom of using medicinal edible plants to strengthen their physical health and prevent disease. They collect many medicinal WEPs for meals and stew them with pork every year during the Dragon Boat Festival [69], which is a holiday adopted from the Han Chinese. Similarly, the Yi, Lahu, and Han people in the Simao area of Yunnan Province, as well as the Zhuang people of Guangxi, have related traditions of eating meals of medicinal roots during the Dragon Boat Festival [70].

Nevertheless, our data on medicinal WEPs also reveal some of the most common diseases and general health concerns that afflict the Liangshan Yi communities (Table 5). In total, taxa from 37 out of the 62 families of WEPs in our study were used for medicine, and, as documented elsewhere [9, 16, 71], for a given species, the medicinal plant parts often differed from those collected for food or other uses (Table 2). For example, *Artemisia capillaris* (UV #3) is an important ritual plant, with its aboveground parts used in almost all cleansing rituals as well as for medicine, but only the tender shoots are used as a famine food. Similarly, the fruit of *Sambucus adnata* (UV #10) is used as a snack, but the aboveground portions have medicinal and ritual uses.

We also documented an interesting preparation method utilized by the Liangshan Yi for four medicinal WEPs, in which plant parts are prepared in fried egg soups for ingestion by the patient. This preparation method is intentional and only used for certain plant parts from particular species to treat known ailments. This appears to be similar to the preparation methods of certain medicinal plants ingested by the Yi people of Chuxiong Prefecture in Yunnan Province [72], but more research is required to understand the significance of the egg preparation technique on the bioactivity of these plant compounds.

Seven out of the ten WEPs with the lowest UVs (Tables 2 and 6) are medicinal plants (with high medicinal value but less cultural significance overall), and none of them is WEPs traditionally used by the Liangshan Yi, except for *Equisetum giganteum* and *Bulbophyllum odoratissimum*. Due to improvements in the transportation and communications infrastructure in the Liangshan region in recent years, the Liangshan Yi now interact with other cultures more frequently and widely than ever before. Consequently, some Yi people have learned about these medicinally valuable species through interacting and trading herbs with the Han Chinese [28].

In recent years, however, the Liangshan Yi have struggled to find certain species of medicinal WEPs as their great economic value has led to commercial exploitation and overharvest, leading to an overall sharp decline in wild populations. For example, commercial acquisition of *Paris polyphylla* across Sichuan was about 300 t in the 1990s, but as the slow-growing wild populations diminished, the commercial collection declined to less than 100 t by 2010 [73]. The overall quality of the collected WEPs has also reportedly declined. Therefore, the Liangshan Yi people started collecting wild seedlings of certain valuable WEPs to plant in their courtyards. As we found, many Liangshan Yi either began collecting seedlings of *Paris polyphylla* from the wild or purchasing seedlings from collecting teams in order to plant in their courtyards (Fig. 7). With 2–3 years of growth, the plants grow large enough to be sold at significantly higher prices, providing a relatively stable cash income supply for the largely subsistence-based farmers. The same situation has been documented among the Lisu people in Nujiang, northwest Yunnan, China, who have similarly begun cultivating medicinal plants with high economic values [74].

This highlights the beginning steps of local domestication for these high-value medicinal edible plants, but this phenomenon also has implications on the biodiversity conservation as well. Essentially, there exists a significant pool of wild-collected germplasm spread out across a relatively extensive network of rural villages with specific knowledge of their provenance. In light of decimated wild populations, these household collections of wild-collected species collectively function as a germplasm bank that could potentially be tapped by conservation organizations wishing to re-establish healthy, genetically diverse, wild populations of these threatened species.

*Rosa roxburghii* presents another example of WEP domestication in Liangshan with conservation implications. Originally harvested from the wild as a snack, *Rosa roxburghii* is now widely planted in many Yi courtyards, where specimens have been selected to produce larger and more evenly maturing fruit. The market price of cultivated *Rosa roxburghii* fruit is now eight to ten times that of the fruit directly collected from the wild [31]. Consequently, further domestication of cultigens may also help alleviate collection pressures on some WEPs.

## Conclusion

Our survey documented 105 WEPs in Liangshan Autonomous Prefecture. The traditional knowledge held about these plants is the result of the accumulated experience by the Liangshan Yi people’s long-term presence living in the local environment. With the rise of functional foods and edible medicinal plants, there is a need to further analyze the nutrition, chemical composition, and bioactivity of the WEPs. For sustainable utilization, some species with high medicinal value but sharp declines in wild populations should be further studied for resource assessment, sustainable use, domestication possibilities, and genetic conservation.

## Data Availability

The dataset supporting the conclusions of this article is included within the article (and its tables).
